# Therapeutic gene silencing of *CKAP5* leads to lethality in genetically unstable cancer cells

**DOI:** 10.1126/sciadv.ade4800

**Published:** 2023-04-05

**Authors:** Sushmita Chatterjee, Gonna Somu Naidu, Inbal Hazan-Halevy, Hanna Grobe, Assaf Ezra, Preeti Sharma, Meir Goldsmith, Srinivas Ramishetti, David Sprinzak, Ronen Zaidel-Bar, Dan Peer

**Affiliations:** ^1^ Laboratory of Precision Nanomedicine, Shmunis School of Biomedicine and Cancer Research, George S. Wise Faculty of Life Sciences, Tel Aviv University, Tel Aviv, Israel.; ^2^ Department of Materials Sciences and Engineering, Iby and Aladar Fleischman Faculty of Engineering, Tel Aviv University, Tel Aviv, Israel.; ^3^ Center for Nanoscience and Nanotechnology, Tel Aviv University, Tel Aviv, Israel.; ^4^ Cancer Biology Research Center, Tel Aviv University, Tel Aviv, Israel.; ^5^ Department of Cell and Developmental Biology, Faculty of Medicine, Tel Aviv University, Tel Aviv, Israel.; ^6^ School of Neurobiology, Biochemistry and Biophysics, George S. Wise Faculty of Life Science, Tel Aviv University, Tel Aviv, Israel.

## Abstract

The potential of microtubule-associated protein targets for cancer therapeutics remains largely unexplored due to the lack of target-specific agents. Here, we explored the therapeutic potential of targeting cytoskeleton-associated protein 5 (CKAP5), an important microtubule-associated protein, with *CKAP5*-targeting siRNAs encapsulated in lipid nanoparticles (LNPs). Our screening of 20 solid cancer cell lines demonstrated selective vulnerability of genetically unstable cancer cell lines in response to *CKAP5* silencing. We identified a highly responsive chemo-resistant ovarian cancer cell line, in which *CKAP5* silencing led to significant loss in EB1 dynamics during mitosis. Last, we demonstrated the therapeutic potential in an in vivo ovarian cancer model, showing 80% survival rate of si*CKAP5* LNPs-treated animals. Together, our results highlight the importance of CKAP5 as a therapeutic target for genetically unstable ovarian cancer and warrants further investigation into its mechanistic aspects.

## Teaser

CKAP5 is important for survival of ovarian cancer cells and might become a unique therapeutic target for other types of cancers.

## INTRODUCTION

Ovarian cancer is one of the most frequent causes of mortality in women, with over 200,000 deaths recorded in 2020, accounting for 4.7% of all cancer mortality among women (*1*). The first-line treatment of ovarian cancer includes maximal surgical resection of the tumor, followed by neoadjuvant chemotherapy with platinum/taxane drugs. Although 60 to 80% of patients initially respond to chemotherapy, 80 to 85% will develop chemo-resistance. Therefore, there is a constant search for new therapeutic targets in ovarian cancer (*2*). Although several drugs targeting poly(adenosine diphosphate–ribose) polymerases, angiogenesis, and folate receptors have shown promising results, none of them were successful in treating ovarian cancer, leaving chemotherapy as first-line treatment for patients with ovarian cancer (*3*, *4*). Many of the chemotherapeutic agents exploit the defects in cell cycle machinery of cancer cells and inhibit cell cycle by blocking mitosis. The majority of available mitosis-targeting chemotherapeutic agents act on tubulin and do not distinguish between healthy and malignant cells. This leads to the development of severe adverse effects including neurotoxicity and myelosuppression (*5*, *6*). Therefore, the challenge is to identify molecular targets that are preferably required for the mitosis of cancer cells.

Cell mitosis is an attractive target for effective treatment of various forms of cancer, with clinical trials mostly focusing on cell cycle kinase inhibitors. However, targeting of nonkinase, cell cycle altering proteins has remained unexplored. One such family of genes includes microtubule-associated proteins (MAPs). MAPs help tubulin molecules to maintain stability as well as dynamics, nucleation, cross-linking, transport, and orientation (*7*). Inhibition of some MAPs such as Tau, MAP4, and MAP2 is known to sensitize cancer cell lines to microtubule-binding chemotherapeutic drugs (*8*–*10*). In addition, microtubule-associated motor proteins, such as Eg5, have shown potential antitumor effects (*11*, *12*). However, MAPs include 200 genes, and their role in tumor development remains largely unexplored.

One such MAP that plays an important role during mitosis is cytoskeleton-associated protein 5 (CKAP5). CKAP5 is widely expressed across various types of cells and plays a very important role in regulating the overall microtubule dynamics in human cells through its microtubule-stabilizing and polymerizing activities. Its absence results in defective mitosis by multipolar spindle formation, reduction in the chromosomal oscillations, reduced tension between kinetochores, and a decrease in spindle microtubule length (*13*–*19*). Despite widespread expression of CKAP5 in various cell types, it can be a promising cancer cell mitosis target due to the vulnerability of cancer cells with high genetic aberrations and cell cycle abnormalities (*20*). In an RNA interference (RNAi) lethality test, potential targets were screened for multiple myeloma cells compared to nonmyeloma cells, and CKAP5 was observed as one of the most differentially vulnerable targets (*21*). Furthermore, *CKAP5* along with four other mitotic genes showed a worse prognosis in patients with non–small cell lung cancer. In addition to that, many of the CKAP5 interacting partners such as Aurora kinase and integrin-linked kinase have shown therapeutic potential in different forms of cancers (*22*, *23*). To this end, lipid nanoparticle (LNP)–mediated small interfering RNA (siRNA) delivery can be harnessed to silence a specific gene, as it is the most advanced nonviral strategy for in vivo nucleic acid delivery (*24*–*26*). In addition, application of LNP-mediated mRNA delivery for SARS-CoV-2 vaccines by Pfizer/BioNTech and Moderna has paved the way for LNP-mediated therapeutics in the clinic. LNPs are multicomponent LNPs that consist of an ionizable amino lipid, helper phospholipid, cholesterol, and poly(ethylene glycol) (PEG) lipid and enable efficient nucleic acid encapsulation, stabilization, and retention in circulation and improve cell penetration.

Here, we screened the effect of *CKAP5* silencing in various solid cancer cell lines and a normal, noncancer epithelial cell line (negative control). We demonstrate that cells with high genetic instability were selectively susceptible to CKAP5 depletion, among them the chemo-resistant NCI-ADR/Res (NAR) ovarian cancer cell line, which was found to be highly sensitive to this cellular manipulation. We observed that *CKAP5* down-regulation led to loss of bipolar mitotic spindle formation, which was substantially more prominent in genetically unstable cancer cells as compared to genetically stable, CKAP5 nonresponsive cancer cells, or normal noncancerous control cells. Further mechanistic investigation through live-cell imaging of *tubulin.GFP* and histone *H2B.mCherry* labeled NAR cells showed that any cell that entered mitosis in the *CKAP5* siRNA–treated group was arrested in metaphase and underwent cell death. Further investigation into the mechanism revealed that tubulin dynamics are halted during mitosis of *CKAP5* knockdown cells. Furthermore, we observed that in vivo treatment with si*CKAP5* LNPs led to significant reduction in tumor volume (*P* < 0.0001) and increased survival in an in vivo xenograft intraperitoneal ovarian cancer model. Overall, our data suggest that CKAP5 is a promising therapeutic target in genetically unstable ovarian cancer.

## RESULTS

### Genetically unstable cancer cells are highly sensitive to siRNA-mediated CKAP5 down-regulation

**Fig. 1.**
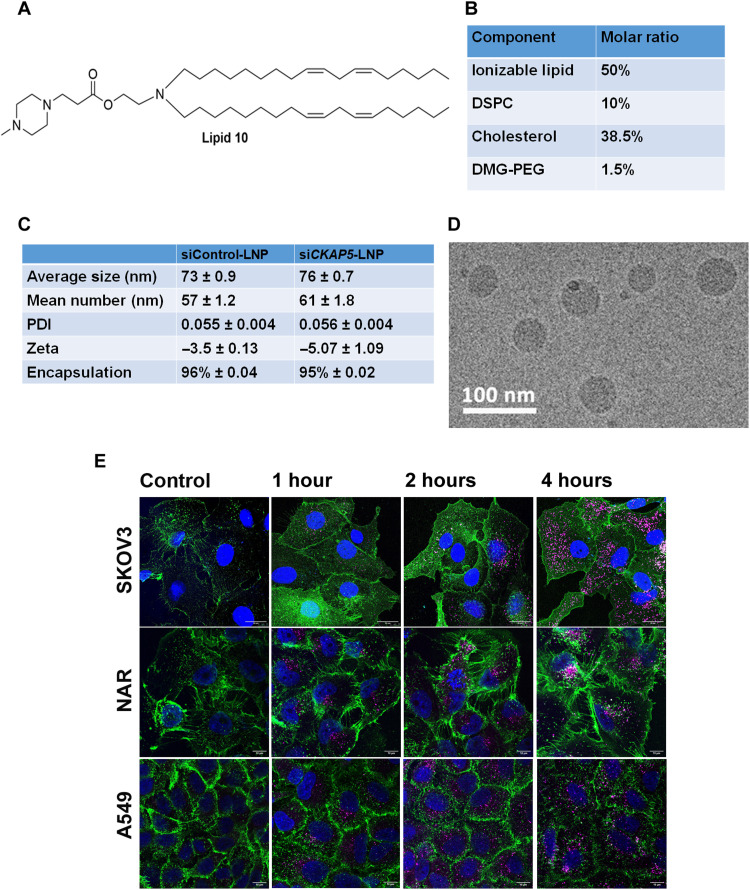
Physicochemical properties of siRNA-encapsulated LNPs and their endocytosis. (**A**) Structure of the ionizable lipid 10 used for LNP preparation. (**B**) Composition of the particles. (**C**) Average size (in nm), zeta potential (in mV), PDI (polydispersity index), and % encapsulation efficiency of the particles. Data are represented as mean ± SEM from three batches of formulations (*n* = 3). (**D**) Cryo-EM image of lipid nano particles. (**E**) Confocal microscopic images of Cy5siRNA-labeled LNP internalization in SKOV3, NAR, and A549 cells. Images are captured at 63×. Cy5 is labeled as magenta, and the cell membrane is stained with rabbit anti-human epidermal growth factor receptor and goat anti rabbit488 antibody. Scale bars, 10 μm.

**Fig. 2.**
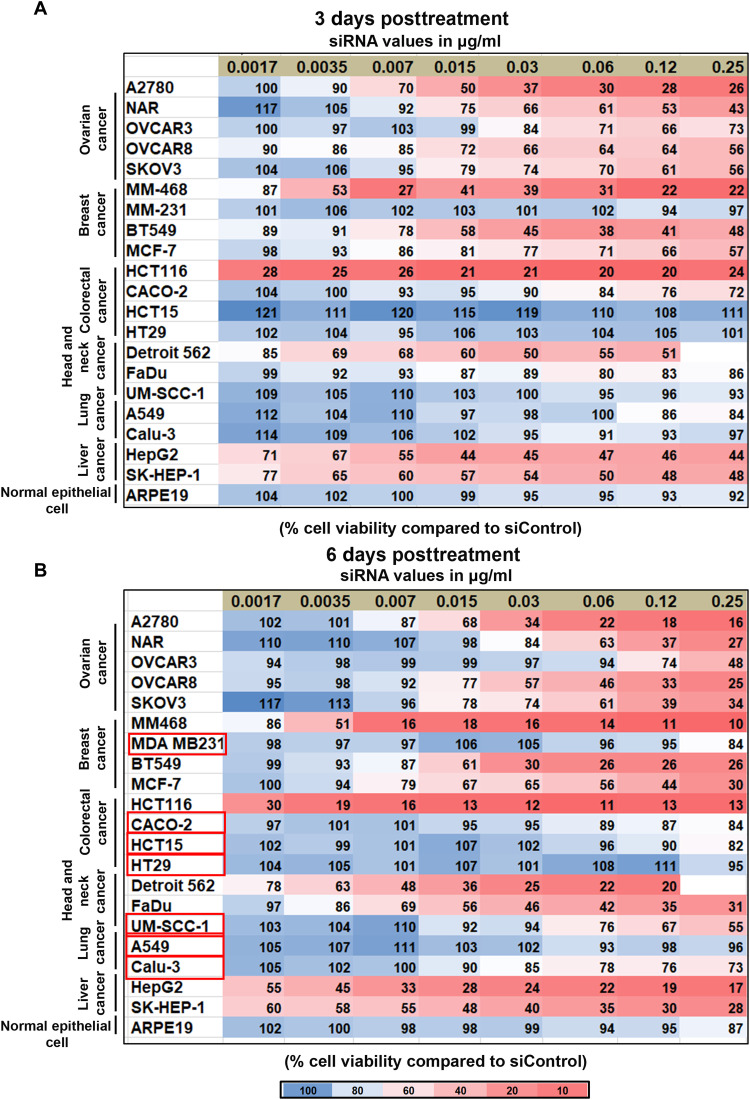
Dose-dependent response of *CKAP5* knockdown across various cancer cell lines. (**A**) Cell viability at 72 hours and (**B**) 6 days post-si*CKAP5* treatment as measured by methoxynitrosulfophenyl-tetrazolium carboxanilide (XTT). Cells were treated with the indicated concentration of si*CKAP5*-LNP and compared with an equivalent concentration of siControl LNP. XTT assay was performed at the indicated time points. Values represent average % cell viability compared to cells treated with an equivalent concentration of siControl LNP. Each experiment was carried out at least three times with three technical repeats each time. Cells that did not show any effect of *CKAP5* knockdown are highlighted with red boxes.

CKAP5 expression across various cancer cell lines has not been well documented. Therefore, we determined the expression of *CKAP5* at the transcript level in a panel of solid cancer cell lines including ovarian (A2780, NAR, OVCAR3, OVCAR8, and SKOV3), breast (MCF-7, MDA MB-231, BT549, and MDA MB-468), colorectal (HCT116, HCT15, HT29, and CACO-2), lung (A549 and Calu-3), liver (HepG2 and SK-HEP1), and head and neck (Detroit562, FaDu, and UMSCC) cell lines. *CKAP5* expression was also evaluated in the noncancer cell line, ARPE19—a normal eye epithelial cell line, which served as a negative control. We detected differential *CKAP5* expression in the tested cell lines, with MDA MB231 and HCT15 showing the highest and lowest *CKAP5* expression, respectively (fig. S1). Since *CKAP5* expression was detected to some extent in all the cell lines tested, we included the entire panel for evaluation of the effect of *CKAP5* silencing on cell viability. To this end, we applied siRNA-encapsulated LNPs. Ionizable lipid is the principal component of LNPs, and in the present study, we used our previously described lipid 10 as the ionizable amino lipid in a defined formulation (Fig. 1, A and 1B) (*27*). LNPs were formed by microfluidic mixing, as previously described (*27*). This produced uniformly sized LNPs with an average size of 70 nm and a partially negative surface charge (Fig. 1C). The size distribution and LNP uniformity were further supported by cryo–electron microscopy data (Fig. 1D). Confocal analysis of particle internalization demonstrated particle uptake as early as 2 hours after transfection, which was further increased after 4 hours (Fig. 1E). After the initial particle characterization, we evaluated the effect of *CKAP5* silencing on cell viability. The effect on cell viability was compared with control siRNA particles at the highest treatment concentration. Cell viability was determined at 3 and 6 days after transfection of the cells with LNPs in the concentration range of 0.015 to 0.25 μg siRNA/ml. Control siRNA particles did not show toxic effects in this range in all cell lines except for the Detroit cell line (fig. S2A). Therefore, further experiments were performed with a lower dose of siRNA (0.12 μg/ml) specifically for this cell line. *CKAP5* silencing was confirmed on the transcript and protein level by quantitative polymerase chain reaction (PCR) and Western blot analysis, respectively (fig. S2, B to E). *CKAP5* silencing resulted in a dose-dependent reduction of cell viability 72 hours posttreatment. Moreover, this effect was more prominent 6 days posttreatment in the majority of the cell lines screened for all the concentrations (Fig. 2, A and B). We determined a threshold of 50% reduction in cell viability for categorizing the cell lines as *CKAP5* knockdown sensitive or resistant. Of the 21 cell lines tested, only 8 were resistant to *CKAP5* silencing, among which was the ARPE 19 cell line, suggesting the nonresponsive nature of normal control cells to *CKAP5* down-regulation (Fig. 2, A and B). Increasing the siRNA concentration up to the limit of nontoxic doses (1 μg/ml) did not increase sensitivity to treatment in almost all cells tested, although efficient silencing was established (fig. S3, A and C). We did not observe any correlation between levels of gene expression or cell doubling time and the response to si*CKAP5* treatment (fig. S3, D and E). Cancer cells are known to harbor genetic instability, which may render them more sensitive to further mitotic damage. Thus, we planned to characterize the genetic abnormalities in the 21 cell lines panel. To this end, we determined micronuclei formation and mitotic damage by 4′,6-diamidino-2-phenylindole (DAPI) and tubulin staining followed by confocal microscopy (fig. S4, C and D). A significant increase in micronuclei formation and mitotic spindle defects was detected in sensitive compared to nonsensitive cell lines (*P* < 0.05), suggesting selective sensitivity of genetically unstable cells to *CKAP5* depletion (fig. S4, A and B).

Among all the cell lines tested for cell viability, A2780, NAR, MM468, BT549, HCT116, SK-Hep-1, and HepG2 were most sensitive to *CKAP5* silencing. All the ovarian cancer cell lines analyzed were sensitive to *CKAP5* down-regulation. Therefore, we focused on ovarian cancer cells for further studies. Specifically, we used the NAR cell line for further studies, as it has been shown to be highly resistant to all chemotherapeutic drugs but detected as sensitive to *CKAP5* silencing in our study.

Overall, our data suggest that *CKAP5* is expressed in all the cell lines included in the present study. *CKAP5* was successfully down-regulated by siRNA-LNPs in all the cell lines. Yet, only cells with high genetic instability were vulnerable to *CKAP5* depletion.

### CKAP5 down-regulation leads to cell cycle arrest and spindle defects in ovarian cancer cells

**Fig. 3.**
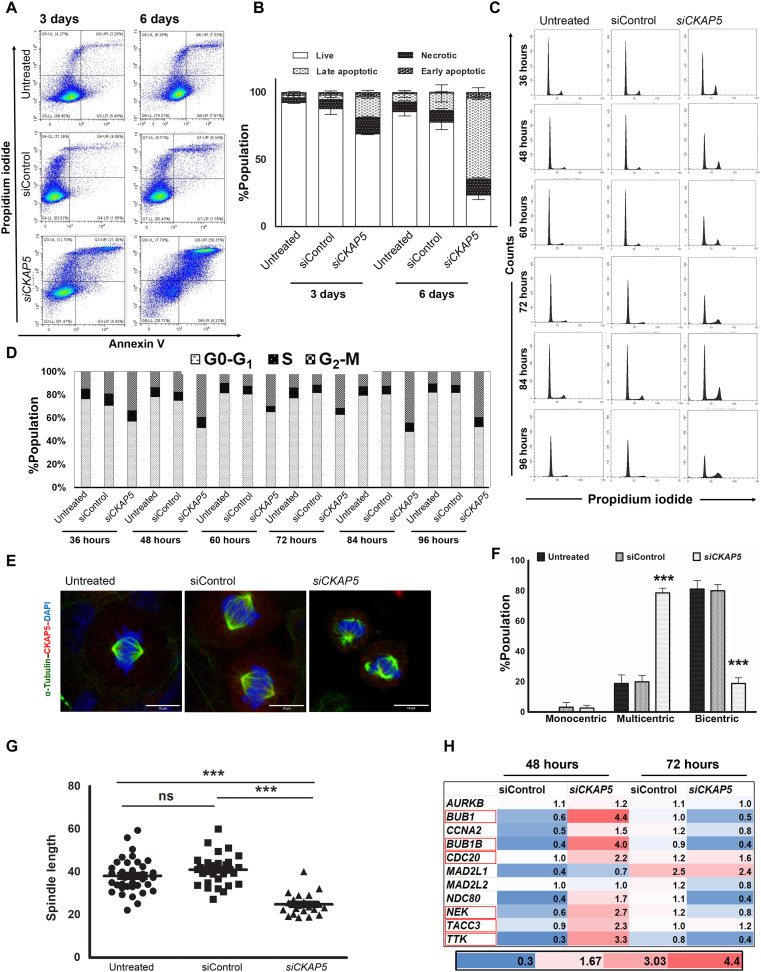
Mechanism of *CKAP5* silencing–mediated cell death in NAR cells. In all the experiments, cells were treated with siControl or si*CKAP5*-LNPs (0.25 μg/ml). (**A**) Dot plot showing apoptotic and necrotic cells in control and treated cells. Cells were harvested at indicated time points and were subjected to flow cytometry after PI and annexin V–APC staining. (**B**) Quantitative measurement of the apoptotic cell death. Data represent average % population ± SEM of three representative experiments (*n* = 2) and analyzed by unpaired *t* test. (**C**) Cell cycle analysis over different time periods post-*CKAP5* knockdown. Cells were harvested at indicated time points and stained with PI after ethanol fixation. (**D**) Quantitative measurement of the % cell population in different phases of cell cycle post-*CKAP5* knockdown (**E**) Confocal microscopic images of spindle formation in control and *CKAP5* down-regulated cells after 48 hours of treatment. Images were captured at 63× with 7× zoom. Scale bars, 10 μm. (**F**) Quantitative analysis of the confocal microscopy images to determine the average number of bipolar spindle formation in control versus *CKAP5* down-regulated cells. Graph shows % cells with indicated spindles in control and treated cells. Data are represented as average ± SEM of 50 events and analyzed by unpaired *t* test. ***P < 0.0001. (**G**) Quantitative comparison of the mitotic spindle length between control and treated cells. The distance between two mitotic poles was counted by the ImageJ analysis tool. Data represent average spindle length ± SEM of 40 events and analyzed by unpaired *t* test. ***P < 0.0001. (H) Reverse transcription PCR analysis of spindle checkpoint genes after 48 and 72 hours of treatment. Data represent average fold change compared to untreated samples from two representative experiments (*n* = 3). ns, not significant.

To study the underlying mechanism of *CKAP5* down-regulation–mediated cell death, an apoptotic assay was performed by propidium iodide (PI)–annexin V staining. Transfecting NAR cells with si*CKAP5*-LNP (0.25 μg/ml) led to 16 and 60% apoptotic cell death, 3 and 6 days after transfection, respectively (Fig. 3, A and B). Furthermore, *CKAP5* silencing arrested 40% of the cells in the G_2_-M phase at 36 hours post-*CKAP5* down-regulation. This arrest was not reversed at any given time point up to 96 hours, when most of the cells already entered apoptosis (Fig. 3, C and D). The effects of *CKAP5* down-regulation on spindle assembly abnormalities have been previously studied mainly in HeLa cells (*13*–*15*). We sought to investigate whether these effects will be detected in NAR cells. To investigate the spindle damage in response to *CKAP5* depletion, cells were stained with tubulin antibody 48 hours post-*CKAP5* silencing. We observed a significant increase in cells arrested in metaphase with multicentric spindle formation (80%) as compared to siControl-LNP–treated (19.9%, *P* < 0.0001) and untreated cells (18.8%, *P* < 0.001) (Fig. 3, E and F). Approximately 20% of control cells also showed multicentric spindle, suggesting endogenous defects in these cells. The detailed analysis of the spindles in *CKAP5*-silenced cells showed reduced spindle axis (~2-fold, *P* < 0.005), reduced spindle density toward the chromosomes, and loss of proper metaphase plate formation (Fig. 3G). The decrease in the density and axis length of the spindle could be due to reduced microtubule polymerization and shorter centrosomal microtubules in the absence of CKAP5 (*13*–*15*). Any mitotic damage results in activation and up-regulation of damage-specific spindle assembly checkpoint genes. Identification of up-regulated genes can provide mechanistic insight into the kind of spindle damage. Therefore, we tested the expression of several spindle checkpoint genes in NAR cells 48 and 72 hours post-CKAP5 silencing. We observed substantial up-regulation in *BUB1*, *BUB1B*, and *TTK* genes 48 hours post-*CKAP5* silencing compared to siControl-LNP–treated cells, which was normalized at 72 hours after silencing whereas there was no effect on the gene expression of *AURKB*, *MAD2L1*, and *MAD2L2* (Fig. 3H). *BUB1*, *BUB1B*, and *TTK* genes are known to function in response to defects in kinetochore-microtubule attachments. Up-regulation of these genes in response to *CKAP5* down-regulation suggests the possibility of a similar disruption mechanism in NAR cells (*14*, *16*). These defects in the cell cycle and spindle assembly formation resulted in increased gamma H2A.X foci formation in the *CKAP5* down-regulated cells, which is a gold standard effect of DNA damage (fig. S5). Overall, we observed that *CKAP5* depletion leads to multicentric spindle formation and G_2_-M cell cycle arrest, followed by apoptosis in NAR cells, which is accompanied by up-regulation of spindle checkpoint genes and gamma H2A.X foci formation.

**Fig. 4.**
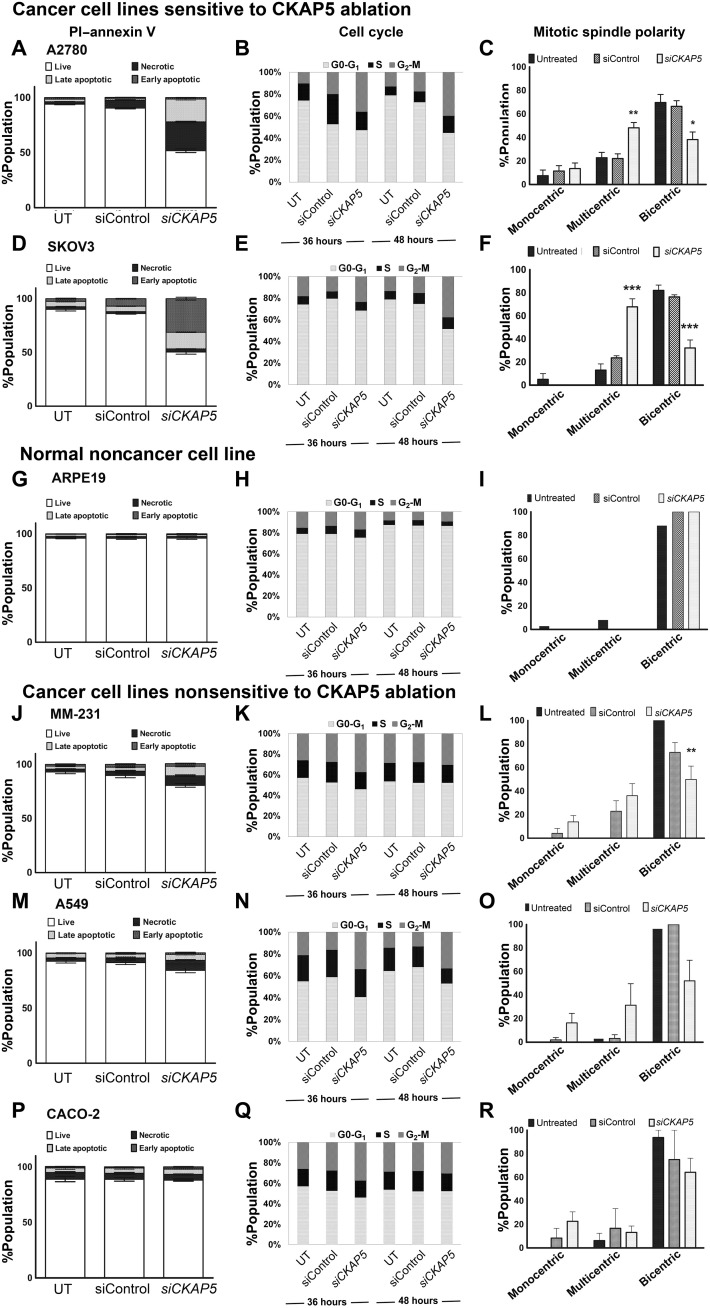
*CKAP5* knockdown mediated effects in various responsive, nonresponsive, and noncancer normal cell lines. A2780 cells representing (**A**) quantitative measurement of apoptotic cell death. (**B**) Cell cycle analysis over different time periods. (**C**) %Population with bipolar spindle formation in control and treated cells. *P = 0.01; **P = 0.005. SKOV3 cells representing (**D**) quantitative measurement of apoptotic cell death. (**E**) Cell cycle analysis over different time periods. (**F**) %Population with bipolar spindle formation in control and treated cells. ***P = 0.0007. ARPE19 cells representing (**G**) quantitative measurement of apoptotic cell death. (**H**) Cell cycle analysis over different time periods. (**I**) %Population with bipolar spindle formation in control and treated cells. MM-231 cells representing (**J**) quantitative measurement of apoptotic cell death. (**K**) Cell cycle analysis over different time periods. (**L**) %Population with bipolar spindle formation in control and treated cells. **P = 0.0062. A549 cells representing (**M**) quantitative measurement of apoptotic cell death. (**N**) Cell cycle analysis over different time periods. (**O**) %Population with bipolar spindle formation in control and treated cells. CACO-2 cells representing (**P**) quantitative measurement of apoptotic cell death. (**Q**) Cell cycle analysis over different time periods. (**R**) %Population with bipolar spindle formation in control and treated cells. In all the graphs, data represents mean ± SEM of three representative experiments (*n* = 2), and statistical analysis was performed by unpaired *t* test. For all the experiments, A2780, SKOV3, and ARPE19 cells were treated with 0.25 μg/ml, and MM-231, A549, and CACO-2 cell lines were treated with 1 μg/ml of siRNA. For analysis of spindle polarity, 50 cell cycle events were counted. Data represent % cells with indicated spindle phenotype.

We next investigated the apoptotic cell death and cell cycle analysis in two other ovarian cancer cell lines, A2780 and SKOV3. For the apoptotic cell death assay, cells were treated with siControl/si*CKAP5*-LNP (0.25 μg/ml) for 96 hours and stained with annexin V–Allophycocyanin (APC) and PI for flow cytometry analysis. A 50% reduction in cell viability was recorded in both cell lines treated with si*CKAP5*-LNPs, while no toxic effects were observed in siControl-LNP–treated group (Fig. 4, A and D, and fig. S6). A2780 cells were observed mainly in the necrotic (26%) and late apoptotic (21%) phases, whereas the SKOV3 cells were observed mainly in the early apoptotic phase (33%). In addition, cell cycle analysis was performed at 36 and 48 hours after incubation with siControl or si*CKAP5*-LNPs. *CKAP5* down-regulation led to G_2_-M cell cycle arrest in both ovarian cancer cell lines tested. In A2780 cells, the arrest was observed 36 hours after transfection and increased even further at 48 hours (1.8-fold, 36 hours; 2.1-fold, 48 hours) (Fig. 4B and fig. S7). In SKOV3 cells, a 1.7-fold increase in G_2_-M arrest was detected at 36 hours post-*CKAP5* down-regulation; however, a substantial increase in G_2_-M arrest (2.5-fold) was observed at 48 hours after transfection (Fig. 4E and fig. S7). Last, these cell lines were evaluated for *CKAP5* down-regulation–mediated defects in spindle assembly formation. In both A2780 and SKOV3 cell lines, *CKAP5* knockdown led to a significant loss in bipolar spindle formation that was associated with increased multipolar spindle formation (52% A2780, *P* value 0.01; 68% SKOV3, *P* value 0.0003) (Fig. 4, C and F, and fig. S8). Both cell lines showed a few multipolar spindles even in siControl-treated and untreated cells (25% A2780 and 20% SKOV3) which is indicative of their damaged genetic makeup. Thus, all the sensitive ovarian cancer cell lines showed G_2_-M cell cycle arrest and increased multicentric spindle formation in response to *CKAP5* down-regulation, which was associated with the loss of cell viability.

We tested all these parameters in the noncancerous, normal epithelial cell line ARPE19. Despite substantial down-regulation of *CKAP5* expression, these cells remained unaffected in terms of cell viability, cell cycle state, and normal bipolar mitotic spindle formation (Fig. 4, G to I).

Next, we analyzed apoptotic cell death of a few nonresponsive cells from different cancer types such as MM-231 (breast cancer), A549 (lung cancer), and CACO-2 (colorectal cancer). A partial, nonsignificant loss of cell viability was recorded in MM-231 and A549 cells (10 and 5% loss, respectively), 96 hours post-*CKAP5* down-regulation (Fig. 4, J and M, respectively). CACO-2 cell viability remained completely unaffected (Fig. 4P), aligning with the methoxynitrosulfophenyl-tetrazolium carboxanilide (XTT) data obtained in the previous assay (Fig. 2, A and B, and fig. S3A). Analysis of cell cycle state in MM-231 as well as CACO-2 cells did not show any notable changes in si*CKAP5*-LNP–treated group at 36 hours as well as 48 hours of incubation (Fig. 4, K and Q, respectively). In the nonresponder A549 cell line, we observed a 2.1-fold increase in G_2_-M arrest at 36 hours that was maintained up to 48 hours (Fig. 4N and fig. S7). Further analysis of spindle formation in response to *CKAP5* down-regulation did not show any notable loss of bipolar spindles in A549 and CACO-2 cells (Fig. 4, O and R, respectively); however, a significant loss (37%, *P* value 0.0003) in bipolar spindle formation was observed in MM-231 cell line which was associated with increased multipolar spindle formation (Fig. 4L). This increase in multipolar spindle formation did not result in apoptotic cell death as observed in Fig. 4A. Monopolar spindles were detected in all nonresponsive cell lines following *CKAP5* down-regulation but not in any of the sensitive cell lines. However, this increase in monopolar spindles was nonsignificant.

Overall, our data suggest that all the sensitive cell lines show a significant (*P* < 0.0001) increase in apoptotic cell death in response to *CKAP5* knockdown which is associated with G_2_-M arrest and increased multipolar spindle formation. Nonresponsive cancer cell lines do not show any apoptotic cell death, despite G_2_-M arrest in A549 cells or increased multipolar spindle formation in MM-231 cells. These characteristics of nonresponder cells are indicative of tolerance of these cells to such mitotic damage. Noncancer normal eye epithelial cell line ARPE 19 did not show any effect of CKAP5 down-regulation on cell viability, cell cycle arrest, and mitotic spindle assembly formation.

### CKAP5-silenced cells show unique cell death mechanisms

**Fig. 5.**
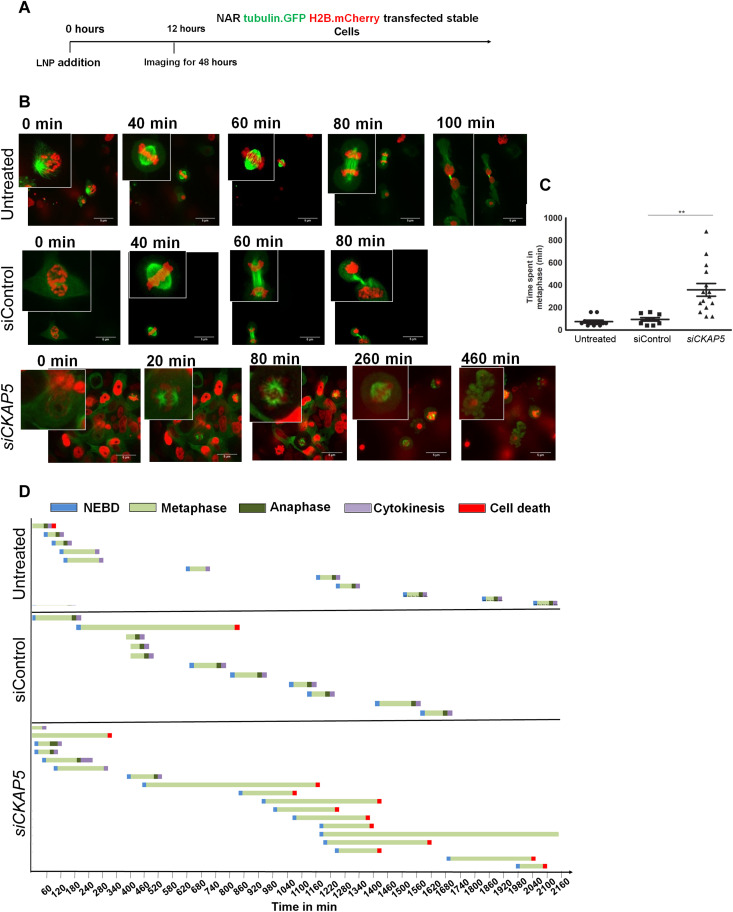
Live cell tracking of *CKAP5*-silenced NAR cells. Cells were labeled with *tubulin.GFP* and histone *H2B.mCherry* to follow the mitotic spindle and nucleus respectively. (**A**) Schematic representation of the experiment performed indicating the timelines of LNP addition and live-cell imaging. (**B**) Representative images to follow cell cycle in NAR cells treated with siControl or si*CKAP5*-LNP. NAR cells labeled with *Tubulin.GFP* and histone *H2B.mCherry* were treated with siControl or si*CKAP5*-LNPs (0.25 μg/ml). Twelve-hour posttreatment live-cell imaging was performed by a spinning disk confocal microscope. Images were captured every 15 min for 48 hours. Scale bars, 5 μm. (**C**) Average time spent in mitosis in different groups as observed with a spinning disk microscope. Data are represented as average time ± SEM from 20 cell cycle events and analyzed by unpaired *t* test. **P = 0.0019. (**D**) Cell fate with respect to time in control and treatment groups. Each bar represents a cell and its fate through different phases of the cell cycle.

To understand the fate of cells with multicentric spindles, we engineered NAR cells for stable expression of *Tubulin.GFP* and *H2B.mCherry* and tracked the spindle formation by live-cell imaging using spinning disk microscopy post si*CKAP5* or a siControl-LNP treatment. We started the live-cell imaging 12 hours posttreatment by capturing images every 15 min until the 60-hour time point (schematically shown in Fig. 5A). Images from the *CKAP5*-silenced group show that in the initial few hours of imaging, all the metaphases observed were completed with a bipolar spindle; however, after 12 hours of imaging, at 24 hours post-*CKAP5* silencing, the majority of the metaphase observed was multicentric (Fig. 5D and movie S3). The average time spent in metaphase was 350 min in *CKAP5*-silenced cells compared to only 80 to 90 min for cells in the siControl and untreated control groups (Fig. 5, B and C). This increased average metaphase time was irrespective of the spindle polarity state, suggesting various other spindle abnormalities in these cells in addition to multicentric spindle formation (movie S3). The majority of the cells that entered mitosis underwent apoptosis (70%) in the *CKAP5*-silenced group. In 6% of the cells imaged, cells divided even with multicentric spindles; however, it was difficult to track the fate of these daughter cells due to time and three-dimensional (3D) plain limitations. The apoptotic cell death occurred via metaphase arrest of cells. None of the cells that were arrested in metaphase could rescue the arrest and enter anaphase, suggesting the vulnerability of these cells to such damage. We observed a few events where cells transitioned between multipolar and bipolar spindle, but in all such events observed, cells remained arrested in metaphase and eventually underwent cell death. In contrast, in the untreated and siControl group, there was only one event of apoptosis, and only a single event of multipolar spindle formation was observed (movies S1 and S2).

**Fig. 6.**
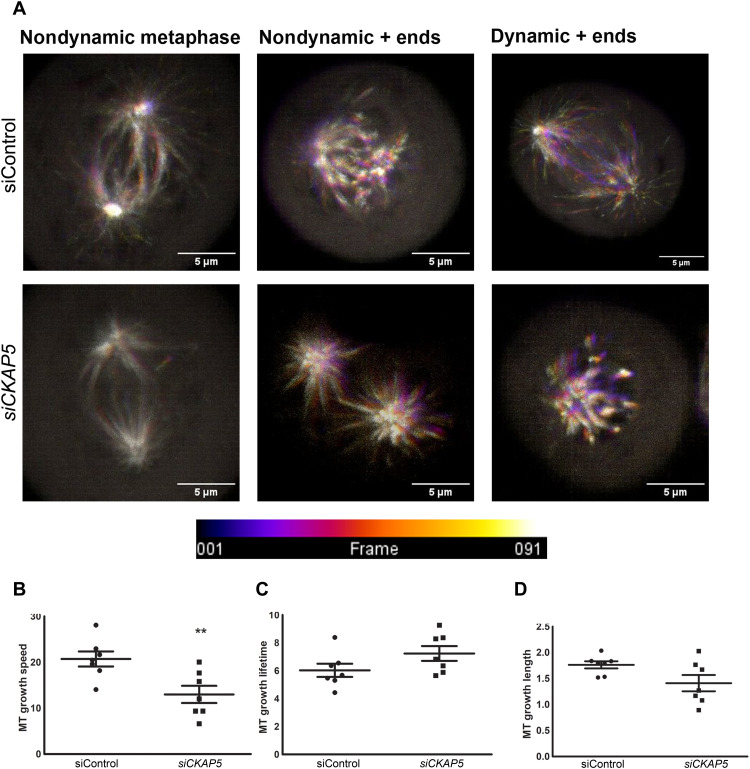
CKAP5 silencing–mediated effects on microtubule dynamics and localization of NAR cells during mitosis. (**A**) Representative phenotypes of EB3 localization in control and *CKAP5*-silenced groups. NAR cells were transiently transfected with *EB3.eGFP* plasmid to label the microtubule + ends. After 12 hours of transfection, cells were further transfected with siControl/si*CKAP5* LNPs, and live-cell imaging was performed after 30 hours of siRNA-mediated silencing. Live cell imaging was performed by a superresolution spinning disk microscope. Images were captured at 100×. Scale bars, 5 μm. Images were captured every second for a period of 90 s. The images represent superimposition images of 91 frames with each frame marked in a specific shade as shown in the time frame scale. Nondynamic + ends superimpose on each other in all 90 frames due to their static nature and represent as white due to superimposition, whereas dynamic + ends do not superimpose in all the 90 frames due to their dynamic behavior and thus appear as a rainbow color. Thus, higher dynamics of tubulin result in a wider range of colors in these representative superimposed images. (**B**) Quantitative representation of the microtubule (MT) growth speed in control and *CKAP5*-silenced group. The live-cell kinetic data obtained from a spinning disk microscope was subjected to Utrack analysis using MATLAB software. **P = 0.0097. (**C**) Quantitative representation of the MT growth lifetime in control- and *CKAP5*-silenced group as measured by MATLAB analysis. (**D**) Quantitative representation of the MT growth length in control and *CKAP5*-silenced group as measured by MATLAB analysis. For all the graphical analysis in (B) to (D), the data are represented as mean ± SEM from 10 mitotic events and statistically analyzed by an unpaired *t* test.

Since even cells with bicentric spindle could not complete metaphase and entered apoptosis in *CKAP5*-silenced group, we investigated the spindle abnormalities in this set of cells. *CKAP5* is a +end tubulin binding protein, so we tracked the +ends of microtubules by EB3 tracking for analysis of tubulin kinetics. Live cell imaging of *EB3.eGFP*-transfected NAR cells through super resolution spinning disc microscopy showed substantial differences in EB3 localization as well as kinetics between si*CKAP5*- and siControl-LNP–treated cells (movies S4 and S5). In the siControl group, the majority of the cells showed EB3.eGFP localization only at the +end tips which were highly dynamic during metaphase (73%). In contrast, the majority of si*CKAP5*-treated cells showed EB3.eGFP localization throughout the spindle, with severely nondynamic spindles (Fig. 6A.). In both groups, there were a few cases where only the +ends were marked but they were not dynamic (Fig. 6A). In the siControl-treated group, only 15% of nondynamic spindle phenotype was observed. Application of the Utrack analysis program of the MATLAB to measure the dynamics as well as lifetime of EB3 comets clearly showed a significant reduction in the microtubule growth rate in the *CKAP5*-silenced group (1.6-fold reduction, *P* value 0.0097) (Fig. 6B). There were small nonsignificant differences in growth lifetime and microtubule growth length in the *CKAP5*-silenced group compared to the siControl group (Fig. 6, C and D). Reduction in spindle length and reduced spindle density in response to *CKAP5* silencing was observed (Fig. 5A) in *CKAP5*-silenced groups irrespective of its dynamic status, further confirming the reduced tubulin density in response to *CKAP5* silencing. Overall, our results clearly demonstrate a dramatic difference of EB3 phenotype as well as kinetics between si*CKAP5*- and siControl-LNP–treated cells.

### LNP biodistribution show localization in liver, spleen, and tumor tissues

**Fig. 7.**
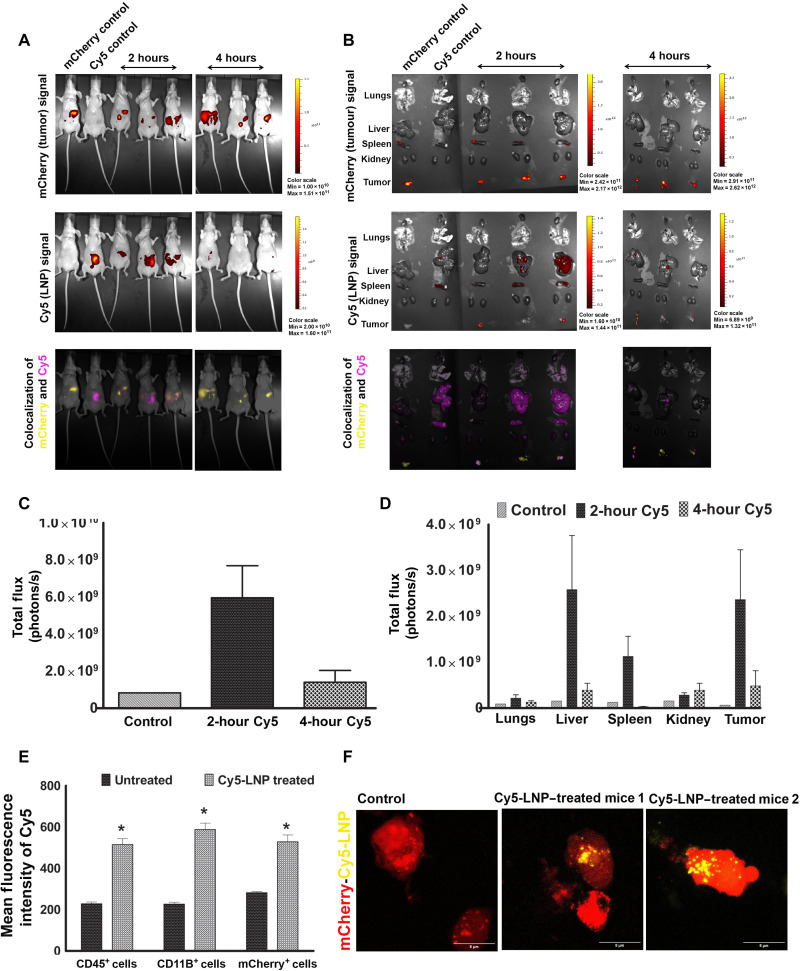
Biodistribution of lipid10-siControlCy5 particles in *mCherry*-labeled NAR xenografted tumor mouse models. (**A**) Cy5, mCherry, and colocalized signal in whole mice after 2 and 4 hours of intraperitoneal injection of siRNACy5 LNPs. *mCherry* NAR-implanted mice were injected with 1 mg kg^−1^ dose of LNP-Cy5 and imaged for Cy5 and mCherry signal after 2 and 4 hours of administration (**B**) Cy5, mCherry, and colocalized signal in extracted organs and tumors after 2 and 4 hours of intraperitoneal injection of Cy5-labeled LNPs. (**C**) Cy5 quantitation in whole mice. Data represent mean Cy5 intensity ± SEM from two representative experiments (*n* = 3). (**D**) Quantitative estimation of Cy5 in extracted organs. Data represent mean Cy5 intensity ± SEM from two representative experiments (*n* = 3). (**E**) Mean fluorescent intensity of Cy5 in tumor and immune cells as quantified by FACS analysis. NAR *mCherry* tumor implanted mice were injected with Cy5-LNP (1 mg kg^−1^), and the tumor was extracted after 2 hours of injection. The tumor was processed for single cells and stained with CD45 APC Fire and CD11B FITC to identify the immune cells and myeloid cells, respectively. Cy5 intensity in these cells together with extracted *mCherry* positive tumor cells was quantified by FACS. Data represent mean fluorescence intensity ± SEM from two representative experiments (*n* = 3), and statistical analysis was performed by unpaired *t* test. *P = 0.0018 (CD45^+^ cells), 0.0035 (CD11B^+^ cells), and 0.004 (mCherry^+^ cells). (**F**) Representative 3D images to show colocalization of Cy5-LNPs with mCherry-positive tumor cells. The image was obtained by 3D z-stacking of tumor cells in a confocal microscope at ×60 magnification with 5× zoom. Scale bars, 5 μm.

After establishing the potential and mechanistic aspects of *CKAP5* silencing, our next aim was to determine the in vivo therapeutic potential. To this end, we first evaluated the biodistribution of LNPs in an intraperitoneal ovarian cancer xenograft mouse model. First, NAR cells labeled with *mCherry* and *luciferase* (*Fluc*) were intraperitoneally implanted in athymic Nude-Foxn1nu mice, and tumor growth was monitored through in vivo imaging system (IVIS) imaging. Next, Cy5-labeled control siRNA-encapsulated LNPs (Cy5-LNPs) were intraperitoneally administered at a 1 mg kg^−1^ dose. As observed in Fig. 7A, in vivo imaging demonstrated a clear Cy5-LNP signal in the peritoneal cavity of mice at 2 hours postadministration, and the majority of the signal was lost at 4 hours postadministration. To better understand the biodistribution and particle localization to the tumor site, we extracted lung, liver, spleen, kidney, and tumor tissues from all the mice and tested the Cy5 and mCherry signal via IVIS imaging. As can be seen in Fig. 7B, Cy5 signal was mainly observed in the spleen, liver, and tumor tissue area at 2 hours postadministration. In the composite image, Cy5 colocalization with *mCherry*-positive tumor cells clearly suggests that the LNPs localize to the tumor tissue. Imaging the tissues at 4 hours post–Cy5-LNP administration showed a weak signal in the liver and tumor tissue area whereas signal from the spleen was lost entirely, suggesting particle clearance from the system. These results were further confirmed by quantitative measurement of the Cy5 signal obtained, confirming LNP localization to tumor tissue in addition to liver and spleen tissues (Fig. 7, C and D).

For further investigation of particle uptake in tumor tissue, the tumor was extracted from untreated and Cy5-LNP–treated mice, 2 hours after particle injection. Tumor tissue was processed to obtain single-cell suspension, followed by staining with anti-CD45 and anti-CD11B to identify mouse immune cells and myeloid cells, respectively. Cells obtained from spleen were used as control for CD45 and CD11B staining. Tumor cells were identified by *mCherry* expression. The Cy5 signal was analyzed in these cell populations through flow cytometry (fig. S9). We observed a twofold increase in levels of Cy5 signal in tumor cells extracted from siRNA-Cy5-LNP–injected mice as compared to untreated tumor-bearing mice (Fig. 7E). In addition, a similar increase in Cy5 intensity was observed in CD45- and CD11B-positive mouse cells. Cy5-positive cells accounted for 12.3% (±0.54), 8.8% (±0.46), and 10.3% (±0.43) of total tumor, CD45-positive, and CD11B cells, respectively (fig. S9). To detect the localization of the Cy5-LNPs within the tumor cells, untreated and particle-treated *mCherry* tumor cells were observed under a confocal microscope where particle localization inside the tumor cells was clearly observed (Fig. 7F).

Overall, Cy5-LNPs are observed mainly in the liver, spleen, and tumor tissue. Through fluorescence-activated cell sorting (FACS) analysis, we detected Cy5 signal in immune, as well as tumor cells, and particle uptake inside the tumor cell was confirmed by a confocal microscope.

### CKAP5 knockdown leads to reduction in xenografted tumor growth and increased survival

**Fig. 8.**
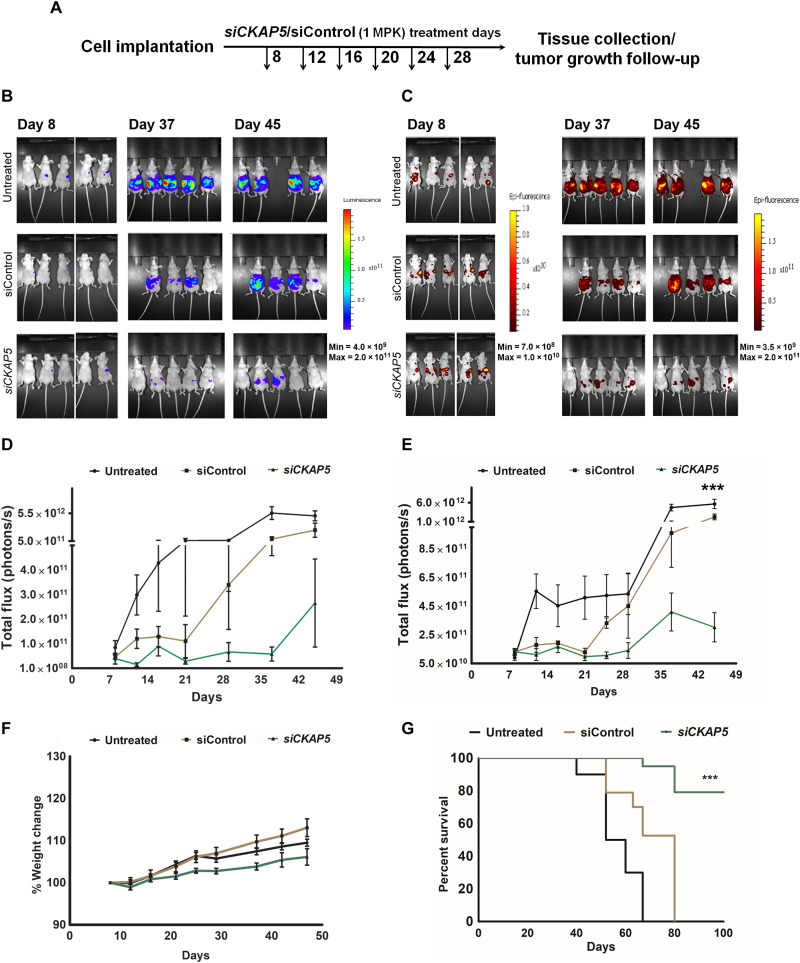
Therapeutic *CKAP5* silencing in NAR-xenografted tumor mice. (**A**) Schematic representation of the experiment timeline. The experiment was repeated twice with *n* = 15 mice per group. Representative data are shown. (**B**) Bioluminescence imaging and (**C**) fluorescence imaging to monitor tumor growth in mice implanted with intraperitoneal administration of NAR mCherry-LUC cells. (**D** and **E**) Quantitative measurement of luminescence and fluorescence signal show tumor growth kinetics in untreated and siControl-LNP–treated mice as compared to si*CKAP5*-LNP–treated mice over 50 days (*n* = 15 mice per group). Data represent average total flux ± SEM from two representative experiments (*n* = 15), and each group was compared with the untreated group by unpaired *t* test. ***P = 0.0009. (**F**) Average weight of mice over days in different treatment groups. Data represent average weight ± SEM from two representative experiments (*n* = 15). (**G**) % Survival data of mice in different treatment groups (*n* = 15 mice per group). Statistical analysis was performed by a log rank test. ***P < 0.0001.

**Fig. 9.**
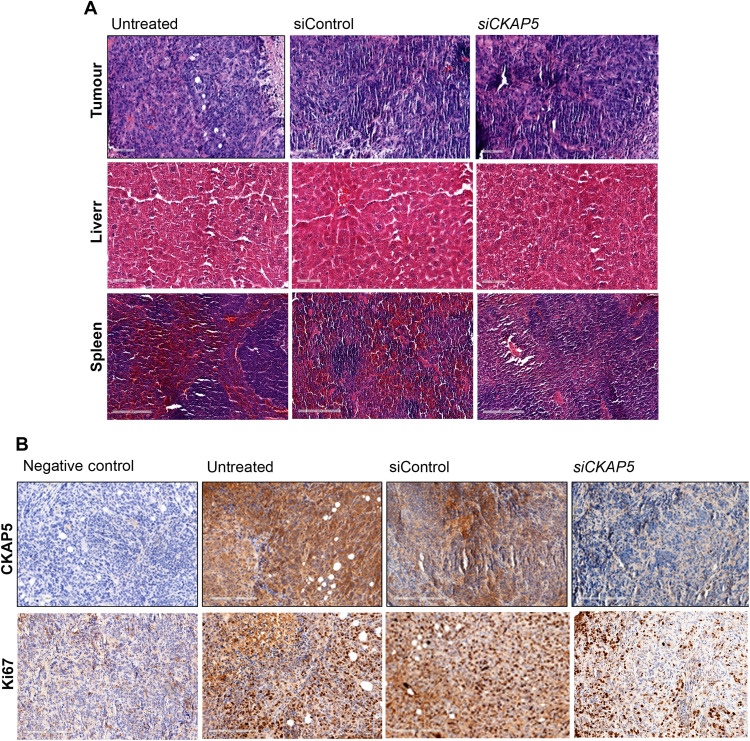
Histological investigation of various tissues extracted from siControl and si*CKAP5*-treated mice. (**A**) H&E staining of liver, spleen, and xenografted tumor tissues isolated from untreated, siControl, and si*CKAP5*-LNP–treated mice. Scale bars in tumor and liver tissue represent 100 μm. Scale bars in spleen tissue represent 200 μm. (**B**) Immunohistochemistry of ovarian cancer peritoneal tumor tissue shows CKAP5 and Ki67 expression in untreated, siControl, and si*CKAP5*-LNP–treated group. Scale bars, 200 μm.

To test in vivo therapeutic gene silencing of *CKAP5*, NAR *mcherry.Fluc* cells were intraperitoneally implanted into mice, and tumor growth was monitored through IVIS imaging. At 8 days postimplantation, mice were randomly grouped into si*CKAP5*-LNP, siControl-LNP, and untreated group (*n* = 20 per group). The mice were treated with 1 mg kg^−1^ dose of si*CKAP5* or siControl LNPs via an intraperitoneal administration. Overall, six doses were administered at 4-day intervals, and tumor growth was monitored by in vivo fluorescence and luminescence imaging (experimental scheme in Fig. 8A). Of 20 mice in each group, 5 mice were sacrificed after six doses of treatment, and the tumor tissues were collected to test the in vivo silencing efficiency of the LNPs and evaluate tissue anatomy by hematoxylin and eosin (H&E). As shown in Fig. 9B, delivery of si*CKAP5*-LNPs resulted in a substantial reduction in CKAP5 expression compared to control groups, suggesting effective silencing of the target gene by LNP-mediated siRNA delivery. Furthermore, Ki67 staining of these tissue samples demonstrated loss of proliferation in the *CKAP5*-silenced group as compared to control groups (Fig. 9B). Liver enzyme analysis was performed, with no indication for particle toxicity in both si*CKAP5* and siControl-LNP treatment (fig. S10). In addition, H&E staining of various organ tissues did not show any differences between si*CKAP5*, siControl, and untreated control groups, suggesting that the particles were not toxic (Fig. 9A). The remaining mice were monitored for tumor growth kinetics and survival.

Tumor kinetic data demonstrated a clear time-dependent increase in mCherry and Fluc signals in untreated and siControl groups, indicating the expected development of the tumor (Fig. 8, B and C). This increase in tumor growth led to ascites development in both the control groups. A total of 50% mice in untreated and siControl-LNP–treated group developed ascites by day 50 and day 67, respectively. Conversely, tumor growth was significantly inhibited in si*CKAP5*-treated animals (sixfold, *P* < 0.0001), and only 3 of 15 (20%) mice showed an increased tumor growth after 42 days (Fig. 8, D and E). A complete eradication of the tumor was observed in five mice, which did not relapse for the entire 90-day experiment period. The decrease in tumor growth resulted in a significantly increased overall survival (80%, *P* < 0.0001) in the si*CKAP5*-treated group; whereas 100% mice developed ascites in untreated and siControl-LNP–treated group by day 67 and day 80, respectively (Fig. 8G). Of the three relapsed tumors in *CKAP5* knockdown mice, two formed ascites after 80 days of implantation. Follow-up of the mice weight suggested an ascites-associated weight increase in siControl-LNPs and untreated groups. The weight increment in the siControl group was similar to the untreated group, suggesting no toxic effects of the siControl-LNPs. In *CKAP5* siRNA–treated mice, weight remained relatively constant up to 40 days, and thereafter, a small increase was detected (Fig. 8F).

Overall, we did not observe any systemic toxicity of siControl/si*CKAP5*-LNP particles as evident from our liver enzyme and H&E data from spleen and liver tissues. LNP-mediated silencing of *CKAP5* showed substantial delay in tumor growth resulting in significantly increased survival in si*CKAP5*-LNP–treated group as compared to control groups (*P* < 0.0001).

## DISCUSSION

Given the numerous chromosomal abnormalities and genetic aberrations in cancer cells, targeting cell cycle for cancer therapeutics has been of great interest for decades (*20*). Presently, tubulin targeting agents are at the forefront of the antimitotic agents. However, there is an urgent need to explore new mitotic targeting agents with better therapeutic index.

MAPs hold great potential as therapeutic targets; however, their potential has not been completely explored due to the lack of target-specific chemical agents (*28*, *29*). RNAi-LNP–mediated perturbation of gene expression holds great promise as a cancer therapeutic strategy due to its high specificity to target genes and minimal toxicity to normal cells. The first LNP-siRNA drug has been approved by the FDA (Food and Drug Administration) in 2018, and many others are already in clinical trials, indicating the potential of this platform (*30*).

CKAP5 is a MAP that plays an important role in mitotic spindle assembly by affecting tubulin functions and helps in centrosomal fragmentation during mitosis. In the present study, we observed that *CKAP5* silencing leads to specific lethality in genetically unstable solid cancer cell lines. Our detailed analysis of 21 cell lines clearly showed a significant innate genetic defects in the susceptible cell lines, which are marked by increased mitotic damage and micronuclei formation (*P* value 0.02). Functional effects of *CKAP5* silencing was evident by G_2_/M arrest and multicentric spindle formation in CKAP5-sensitive ovarian cancer cell lines, NAR, A2780, and SKOV3. In addition, viability of noncancer, normal control epithelial cell line ARPE19 was not affected in response to *CKAP5* knockdown. Further analysis in nonresponder cells for understanding the underlying mechanisms showed an increase in the multipolar spindle formation and cell cycle arrest in response to *CKAP5* down-regulation but was not associated with cell death. These results suggest that CKAP5-nonresponder cancer cells are more tolerant to this damage, possibly due to a more stable innate genetic makeup. Our data clearly show that si*CKAP5*-mediated cytotoxicity is specific only for cells with high chromosomal abnormalities, which is absent in normal noncancerous cells, highlighting CKAP5 as a unique and attractive target.

Genetic instability in ovarian cancer is very well known, and the underlying mechanisms include presence of supernumerary centrosomes, elevated microtubule dynamics, and replication stress (*31*). All the ovarian cancer cells tested in our study demonstrated sensitivity to *CKAP5* silencing, and among them, the chemoresistant NAR cell line was also highly responsive to lethal effects of *CKAP5* knockdown. Therefore, further mechanistic studies were performed in the NAR cell line.

Follow-up of metaphase-arrested NAR cells post-*CKAP5* silencing via live-cell imaging showed that all the cells that entered mitosis could not complete the process in the absence of *CKAP5*, irrespective of spindle abnormality, which resulted in cancer cell death. Furthermore, we sought to elucidate the mechanism of cell death in cells containing proper bicentric spindles. Our live-cell EB3 kinetic data clearly showed a significant (*P* value 0.0097) reduction in mitotic tubulin dynamics in these cells which is different from the kinetic data observed with HeLa cells in previous reports, suggesting the NAR cell–specific role of CKAP5 in microtubule dynamics during metaphase.

To evaluate in vivo biodistribution, efficacy, and silencing, we used an intraperitoneal xenografted ovarian cancer model that recapitulates ovarian cancer metastasis through different peritoneal organs, as observed in the clinic. Biodistribution analysis by IVIS imaging of Cy5-LNPs clearly showed particle localization to the tumor site, as well as liver and spleen area. The prolonged retention and localization of the particles in the tumor area were possibly a result of enhanced permeability and retention effect, which resulted in higher uptake of LNPs by the tumor cells due to leaky vasculature of tumor tissue. Although some signal was detected 4 hours postadministration, the majority of the particles were cleared off from the system by that time point, representing classic kinetic behavior of siRNA-loaded LNPs.

Most cells uptake very few particles, yielding a very low signal intensity that is hard to detect by in vivo fluorescence imaging systems. Further analysis of extracted *mCherry*-positive tumor cells by flow cytometry demonstrated Cy5 signal in 12% of tumor cells. It is possible that this low frequency of Cy5 cells is due to the limit of signal-to-noise ratio of FACS and imaging devices, which underrepresents the actual number of Cy5-positive cells. Administration of six doses of 1 mg kg^−1^ si*CKAP5*-LNPs most likely increased the frequency of transfected tumor cells, resulting in efficient knockdown and increased survival in si*CKAP5*-LNP–treated group (Figs. 8, C, D, and F, and 9B). Overall, these results suggest that in the reported settings, no particle targeting moiety is necessary for delivery, as LNPS are injected intraperitoneally and are in the direct vicinity of relevant organs.

In the present study, we used intraperitoneal injection for LNP delivery, as superior effects of this route have been reported in past studies (*32*). Our experiment’s endpoint was ascites formation, a signature characteristic of advanced-stage ovarian cancer in clinical settings. We observed a significant reduction in tumor growth, which was corroborated by increased survival in *CKAP5*-silenced mice (*P* < 0.0001). Achieving such substantial therapeutic efficacy by silencing a single gene by siRNA is very impressive since RNAi silencing is transient and most of the genes have redundant functions, and tumor growth inhibition is expected to require the silencing of several genes simultaneously. A mild, nonsignificant lag in tumor growth of siControl-LNP–treated group was observed during early phase of tumor growth. However, the difference in the signal intensity between control siRNA-treated and the untreated groups was nonsignificant (*P* value 0.3155). The delayed growth of the tumor cells can be explained by the heterogeneity of the tumor growth during the early establishment of the tumor cells. In addition, the administration of any synthetic material such as LNPs may affect cell metabolism without imposing any cytotoxicity (*33*), which could also account for the lag in tumor growth. Conversely, no differences in tumor growth were observed in later stages of tumor development, between untreated and siControl-treated groups. In contrast, si*CKAP5*-treated mice exhibited significant reduction in tumor growth (*P* < 0.0001).

Overall our efficacy results clearly show the vulnerability of cancer cells to *CKAP5* silencing in vitro as well as in vivo. For translation of CKAP5 as a therapeutic target for genetically unstable cancer cells in the clinic, in future, it will be important to check its therapeutic efficacy in syngeneic and in patient-derived xenograft mouse models.

RNAi-mediated gene silencing is a transient process and usually requires parallel down-regulation of several genes responsible for redundant functions, to inhibit tumor growth. Here, we report substantial therapeutic efficacy by silencing a single gene by siRNA, emphasizing the potency of si*CKAP5*-LNPs. Overall, our efficacy results clearly show the vulnerability of cancer cells to *CKAP5* silencing in vitro as well as in vivo.

Since ovarian cancer is diagnosed mostly at late stages, it will be of great interest to study the effects of *CKAP5* silencing in late-stage ovarian cancer metastasis. In addition, given the reported ability of cancer cells to develop resistance against targeted and nontargeted therapy, it would be of great relevance to explore combination therapies for *CKAP5* siRNA–mediated efficacy. Now, application of LNP-mediated siRNA delivery is mostly limited to the hepatic tissues in clinical settings. Our biodistribution and efficacy data clearly show that even without targeting, our particles reached the tumor cells efficiently and demonstrated excellent retention, as evident from effective silencing and efficacy data. In the future, it will be interesting to recapitulate this in other cancers that metastasize and localize to the intraperitoneal cavity such as colorectal, pancreatic, and liver cancers.

## MATERIALS AND METHODS

### Cell lines

All the cell lines were maintained in 5% CO_2_ incubator and subcultured according to American Type Culture Collection (ATCC) guidelines. Table S1 represents the medium and supplements used for various cell lines used in the study. All the cell lines except NAR and A2780 were purchased from ATCC and are maintained in laboratory according to the required culture and freezing conditions. The A2780 cell line was purchased from Sigma-Aldrich and are maintained in laboratory according to the required culture condition. The NAR cell line was a gift from R. Margalit.

### Cell culture

All the cell culture medium, fetal bovine serum, phosphate-buffered saline (PBS), trypsin, and related products were obtained from Biological Industries/Sartorious.

### LNP preparation

Cholesterol and 1,2-Distearoyl-sn-glycero-3-phosphocholine (DSPC) were obtained from Avanti Polar Lipids, USA. The ionizable lipid 10 was synthesized according to the previously described method (*1*). Chemically modified siRNAs against CKAP5, siControl, and Cy5-NC5 were obtained from IDT.

LNPs were prepared by using microfluidic micro mixture (Precision NanoSystems, Vancouver, BC) as previously described (*34*). Briefly, one volume of lipid mixtures (ionizable lipid, DSPC, cholestrol, and Dimethylglyoxime-PEG at 50:10:38.5:1.5 mole ratio) in ethanol and three volumes of siRNA [1:16 (w/w) siRNA to lipid] containing acetate buffer solutions were mixed through the micro-mixer at a combined flow rate of 12 ml min^−1^. The resultant mixture was dialyzed against PBS (pH 7.4) for 16 hours to remove ethanol. For Cy5-labeled particles, 50% Cy5-labeled nontargeted siRNA was used, and the amount of siRNA-encapsulated was calculated by ribogreen assay (Quant-it Thermo Fisher Scientific R11490).

### Size and charge measurement of LNPs

The size and zeta potential of LNPs-siRNA were measured by dynamic light scattering using Malvern Nano ZS Zetasizer (Malvern Instruments Ltd., Worcestershire, UK). Size and zeta potential measurements were performed in PBS (pH 7.4) and water, respectively.

### XTT

Two thousand to 3000 cells were seeded in a 96-well plate. After 24 hours, cells were treated with mentioned concentration of control siRNA or *CKAP5* siRNA. Cells were incubated with the treatment for 3 or 6 days, and at the end of treatment, XTT was performed (Sartorious, catalog no. 20-300-1000), and plates were read in a plate reader (BioTek Synergy HTX Multimode Reader) at a wavelength of 450 and 630 (background). Live cell population was quantified as compared to control siRNA-treated cells.

### Real-time PCR

For standard curve of *CKAP5* mRNA, gene block was ordered from IDT. Standard curve was made in a concentration range of 10^3^ to 10^6^ copies of gene block. Real-time PCR was performed by the SYBR green method (Quantabio PerfeCTa SYBR green Fast mix) using Step one plus from applied Biosystems. Primers used were specific to gene block, and data obtained were used to plot a graph as Ct (threshold cycle) value versus gene copies. RNA was isolated from cells using an RNA extraction kit (EZ-RNA Total RNA Isolation SKU20-400-100, Biological Industries). RNA was measured in NanoDrop, and 1 μg of RNA was used to prepare cDNA by a quanta cDNA synthesis kit (qScript cDNA synthesis kit). Real-time PCR was performed using the same primers used for gene block, and absolute copies of mRNA were calculated on the basis of the equation obtained from standard curve.

To validate knockdown efficiency, cells were seeded in a six-well plate and treated with siControl/si*CKAP5* LNPs for 48 hours at a concentration of 0.25 μg or 1 μg/ml. RNA isolation, cDNA preparation, and real-time PCR were performed as mentioned before.

For testing the spindle checkpoint genes up-regulated in response to *CKAP5* knockdown, cells were treated with siControl/si*CKAP5*-LNPs (0.25 μg/ml) for 48 and 72 hours. The rest of the procedure was performed as mentioned before. Primer sequence is given in table S2.

### Western blotting

The Western blot apparatus and membranes used were from Bio-Rad. Cells were washed with 1× PBS, and protein was isolated with radioimmunoprecipitation assay buffer (Millipore, catalog no. 20-188) containing protease and phosphatase inhibitors (Cell Signaling Technology, 58715). Protein was measured with a Bicinchoninic acid assay (BCA) protein detection kit (Pierce BCA Protein Assay Kit, Thermo Fisher Scientific, catalog no. 23225), and samples were read in a plate reader (BioTek Synergy HTX Multimode Reader). Protein quantity was measured with a standard curve made in the range of 0.03- to 2-μg protein. Sixty-microgram protein was loaded with sample buffer, containing bromophenol blue and β-mercaptoethanol. Samples were boiled with sample buffer containing 5% β-mercaptoethanol at 100°C for 5 min before loading in 10% SDS gel. Proteins were transferred from gel to the nitrocellulose membrane by the wet transfer method for overnight at 30 volts. Next, the nonspecific sites were blocked with 3% BSA (MP Biomedicals, 160069) in Tris buffered saline with tween-20 (TBST) for 1 hour at room temperature, and the membrane was incubated with primary CKAP5 (1:500) (Abcam, 236981) or α-tubulin antibody (1:5000) (Sigma-Aldrich, 29026) overnight at 4°C. Primary antibody was diluted in 3% BSA made in TBST (0.01% Tween 20) with sodium azide (0.05%). After primary antibody incubation, membrane was washed with TBST (0.1% Tween 20) three times, each for 7 min. Further, the membrane was incubated with respective horseradish peroxidase (HRP)–labeled secondary antibody (1:10,000; The Jackson Laboratory) diluted in blocking buffer for 1 hour. Membranes were again given 7 min wash with TBST three times. Next, the substrate for HRP was added (Pierce ECL Western Blotting Substrate, catalog no. 32209), and chemiluminescence intensity was captured in the Syngene PXi Image Analysis system.

### Confocal imaging of fixed cells

Cells (ATCC) were seeded on coverslip in a 24-well plate and incubated with siControl-Cy5–labeled LNPs for mentioned hours. Cells were then washed with PBS twice and fixed with 4% paraformaldehyde (PFA) for 30 min at room temperature. Cells were washed twice with PBS and blocked with 2% BSA for 1 hour at room temperature. Cells were further incubated with rabbit anti-human epidermal growth factor receptor antibody (Bio-Rad, clone, ICR10) at a dilution of 1:500 for 1 hour followed by secondary rabbit Alexa Fluor 488 antibody at a dilution of 1:800 (The Jackson laboratory), and the nucleus was stained with Hoechst. Cells were mounted with Mowiol, and imaging was performed using a Leica SP8 confocal microscope.

For tubulin staining, cells were treated with siControl/si*CKAP5*. After 48 hours of treatment, a 24-well plate was centrifuged at 1200 rpm for 5 min. Cells were washed with PBS and fixed with chilled methanol at −20°C for 10 min. For permeabilization of nucleus, cells were further incubated with 0.1% Triton X-100 in chilled methanol and incubated at −20°C for another 10 min. Cells were washed thrice and blocked with 2% BSA in PBS for 1 hour at room temperature. Cells were incubated with tubulin antibody (Sigma-Aldrich, 29026) at a dilution of 1:1000 overnight at 4°C. The next day, cells were washed thrice with PBS and incubated with mouse secondary Alexa Fluor 488–labeled antibody (Thermo Fisher Scientific, A21121) at a dilution of 1:1000 for 1 hour at room temperature. Cells were washed twice, and nucleus was stained with Hoechst (0.01 μg/μl) for 5 min. Cells were washed twice, and coverslips were mounted with Mowiol and slides were stored in dark.

For screening of mitotic assembly abnormalities in our panel of cell lines, cells were synchronized in G_2_-M phase by sequential treatment of thymidine (2 mM, 16 hours), 4 hour-release, and nocodazole (0.1 μg/ml, 8 hours), which was followed by cell fixation, permeabilization, and tubulin 640 antibody (BioLegend, 627908) staining. Fifty mitotic events were captured for each cell line in a Leica SP8 confocal microscope at 63×.

For γH2A.X foci assay, cells were fixed with 4% PFA for 30 min at room temperature and permeabilized with PBST (0.05% Triton X-100). Cells were incubated with γH2A.X primary antibody (gift from Y. Shiloh laboratory) (1:500) for 2 hours at room temperature. Further, cells were incubated with secondary 488 antibody at a dilution of 1:1000 for 1 hour at room temperature, and nucleus was counter stained with Hoechst. All fixed cells were imaged with a Leica SP8 confocal microscope at 63× with the relevant laser wavelengths.

### Stable cell line generation

NAR cells were seeded in a six-well plate and transfected with *Tubulin.GFP* plasmid (Addgene plasmid #56450) with Jet Optimus transfection reagent (Polyplus #101000025). Enhanced green fluorescent protein (EGFP)–tubulin 6 was a gift from M. Davidson (Addgene plasmid #56450; http://n2t.net/addgene:56450; RRID:Addgene_56450) (*35*). After 48 hours, cells were sorted for GFP-positive cells in FACSAria. The cells with the highest GFP signal were selected for sorting. The cells were cultured with G418 (0.5 mg/ml) (Sigma-Aldrich, # 1720) for 2 weeks and resorted for GFP-positive cells. Next, cells were transfected with *H2B.mCherry* plasmid, gifted by R. Benezra (Addgene plasmid #20972; http://n2t.net/addgene:20972; RRID:Addgene_20972) (*36*), and sorted after 48 hours for *GFP* and *mCherry* double-positive cells. Cells were cultured in G418 for 1 week, and single-cell dilution was made in a 96-well plate. Single-cell clones were grown for 15 days with medium change every 3 days. Cell clones were monitored under a Nikon Eclipse Ti fluorescent microscope, and few of the clones with high *tubulin.GFP* and high *H2B.mCherry* were amplified and monitored. One of the best clones was used for further live-cell experiments.

### Live cell imaging to follow cell cycle

Twenty thousand NAR-*tub.GFP-H2B.mCherry* cells were seeded in a black glass-bottom 24-well plate in a phenol red-free RPMI media. The next day, cells were treated with siControl or si*CKAP5*-LNP. Live cell imaging was performed after 12 hours of incubation with the respective siRNA-LNP. Images were captured every 15 min for 48 hours. Cells were imaged using an Andor revolution spinning disk confocal microscope (Andor, Belfast, Northern Ireland) at confocal laser wavelengths of 488 and 543 nm. The imaging setup consisted of an Olympus inverted microscope with an oil-immersion Plan-Apochromatic 60× objective numerical aperture (NA) = 1.42 (Olympus, Tokyo, Japan) and an Andor iXon Ultra Electron Multiplication Charge Coupled Device camera (Andor, Belfast, Northern Ireland). For each time point, 8 to 12 *z*-planes of 1-μm thickness were taken. Z-stack merging and movie construction were performed with ImageJ Fiji software.

### Live cell imaging to follow EB3 kinetics

NAR cells were seeded in a glass-bottom 35-mm plate. Cells were transfected with *EB3.eGFP* plasmid (gifted by R.Z. Bar laboratory) by Jet Optimus transfection reagent. After 8 hours of transfection, medium was changed, cells were further transfected with siControl/si*CKAP5*-LNPs, and images for EB3 dynamics were captured with a spinning disk microscope after 36 hours of incubation. Ninety-one images were captured at an interval of 1 s each. The 488 laser wavelength was used with a 100× oil NA 1.45 objective.

### MATLAB analysis of the EB3 kinetic data

EB3 dynamics was quantified by using the utrack MATLAB analysis method as mentioned in the protocol.

### Cell cycle and apoptotic cell death assay

For cell cycle analysis, 5 × 10^4^ cells were seeded in a 24-well plate. Cells were treated as mentioned and collected after 48 hours of LNP transfection. The cells were washed with ice-cold PBS and fixed with 70% ethanol overnight. Further, the cells were washed twice with cold PBS, incubated with ribonuclease for 10 min at 37°C in 100 μl of PBS, and incubated with PI (50 μg/ml) for 30 min at room temperature. Fluorescence was measured by flow cytometry (Cytoflex, Beckman Coulter, USA). Cell viability was evaluated by flow cytometry using annexin V–APC (BioLegend, 640941) and PI as recommended by the manufacturer. Data from at least 5 × 10^4^ cells were acquired using CytoFLEX and the CytExpert software (Beckman Coulter, USA). Analysis was done with the CytExpert software.

### Animal experiments

All animal protocols were approved by the Tel Aviv University Institutional Animal Care and Usage Committee and in accordance with current regulations and standards of the Israel Ministry of Health. All animal experiments were performed in a double-blinded fashion. Mice were randomly divided into different treatment and control groups in a blinded fashion at the beginning of each experiment.

### In vivo biodistribution assay

Eight-week-old athymic Nude-Foxn1nu mice (Envigo, Rehovot, Israel) were injected with 5 × 10^6^-NAR mCherry-LUC cells intraperitoneally. Tumor growth was monitored every fourth day by fluorescence and bioluminescence imaging. For biodistribution analysis, on eighth day of tumor implantation, mice were intraperitoneally injected with 1 mg per kilo of Cy5- labeled siControl LNPs (50% Cy5-labeled RNA). Mice were imaged for mCherry and Cy5 fluorescence after 2 and 4 hours of Cy5-LNP treatment. Further, mice were euthanized, and various organs such as lungs, heart, liver, spleen, kidney, and tumors were extracted and imaged in IVIS using spectral unmixing method to separate the mCherry and Cy5 signal. Fluorescence intensity of Cy5 as well as mCherry was analyzed using the Living Image software (PerkinElmer Inc.). For this, scales from all images were brought to the same level. Region of interest of same size was created for control and treated groups, and fluorescent signal was measured as total flux and plotted accordingly.

### Flow cytometer analysis for identifying the cell-specific LNP uptake

*mCherry*-labeled tumor was extracted from untreated as well as Cy5-labeled siCcontrol-LNP–treated mice. Tumor tissue was processed with collagenase enzyme and passed through 70-μl strainer to obtain single cells. The cell suspension was subjected to red blood cell lysis buffer, centrifuged and collected in FACS buffer, and stained with specific antibody for CD45 and CD11B. Cells were stained with DAPI to identify live cells. Spleen tissue from untreated mice was also extracted and processed, stained in the similar manner, and used as control for single antibody staining. Flow cytometry was performed with respective channels, and gates were set as shown in fig. S9.

### In vivo efficacy assay

Eight-week-old athymic Nude-Foxn1nu mice (Envigo, Rehovot, Israel) were injected with 5 × 10^6^-NAR *mCherry-LUC* cells intraperitoneally. Tumor growth was monitored every fourth day by fluorescence and bioluminescence imaging. Mice that showed tumor formation on eighth day were randomly distributed into three groups for untreated, siControl-treated, and si*CKAP5*-treated groups. Mice were intraperitoneally injected with 1 mg per kilo of siControl or si*CKAP5* LNPs. Mice were imaged for mCherry fluorescence as well as firefly luminescence every fourth day of the treatment. Overall, six doses were given at an interval of 4 days each. At the end of the treatment, five mice from each group were sacrificed to isolate tumor tissues for testing *CKAP5* silencing and H&E staining. The rest of the mice were monitored to test survival, and weight was checked every fourth day. Mice were sacrificed once ascites formation was observed. Fluorescence as well as bioluminescence imaging was analyzed using the Living Image software (PerkinElmer Inc.).

### Immunohistochemistry

Xenograft tumors harvested 72 hours after the last LNP injection were fixed in 10% formalin solution overnight at room temperature, washed and preserved in 70% ethanol solution at 4°C. Formalin-fixed paraffin-embedded slides were prepared and stained with H&E by the Multistainer Leica ST5020 instrument (Leica, USA). Immunohistochemistry was performed by the Leica Bond III instrument (Leica, USA) with acidic antigen retrieval for 20 min, and tissues were stained with CKAP5 antibody (Abcam, ab108349) at a dilution of 1:400. For Ki67 staining, basic antigen retrival was performed for 20 min, and slides were stained with Ki67 antibody (Leica, catalog no. PA0118). The image of slides were captured by a slidescanner microscope (Aperio, Leica).

## Notes

View/request a protocol for this paper from *Bio-protocol*.
