# Supplementary material for: Therapeutic gene silencing of CKAP5 leads to lethality in genetically unstable cancer cells
Source: Sci Adv. 2023 Apr 5;9(14):eade4800. doi: 10.1126/sciadv.ade4800 (PMC10075965; doi:10.1126/sciadv.ade4800)
Supplement: Supplementary file 1 — Figs. S1 to S10 Tables S1 to S4 Legends for movies S1 to S5 [file sciadv.ade4800_sm.pdf]

Science Advances
AAAS

Supplementary Materials for

Therapeutic gene silencing of *CKAP5* leads to lethality in genetically unstable cancer cells

Sushmita Chatterjee *et al.*

Corresponding author: Dan Peer, peer@tauex.tau.ac.il

Sci. Adv. **9**, eade4800 (2023)
DOI: 10.1126/sciadv.ade4800

The PDF file includes:

Figs. S1 to S10
Tables S1 to S4
Legends for movies S1 to S5

Other Supplementary Material for this manuscript includes the following:

Movies S1 to S5

Supplementary Figure 1

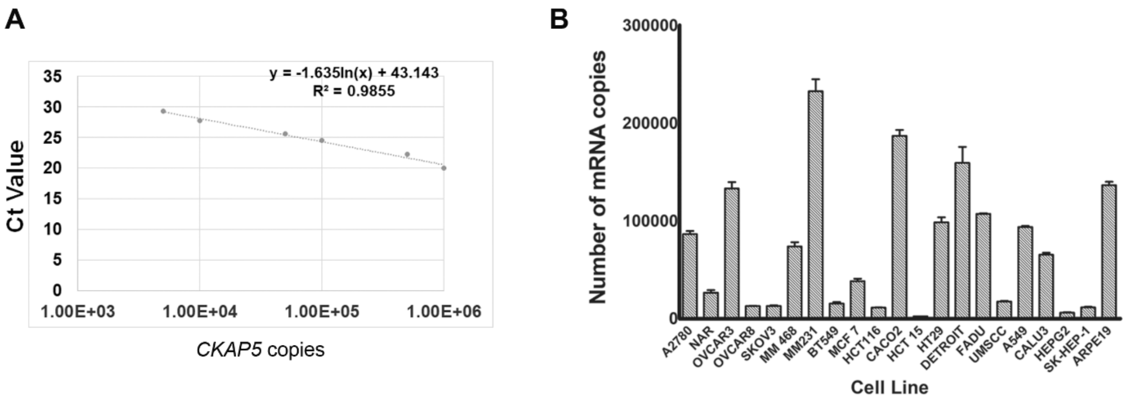

CKAP5 expression across various solid cancer cell lines **A.** Standard curve of *CKAP5* gene block. *CKAP5* gene block was diluted in various concentrations in the range of 10^3 to 10^6 and real-time PCR was performed. CT values obtained were plotted against respective mRNA copies. **B.** mRNA copies of *CKAP5* across various cell lines based on *CKAP5* gene block standard curve. RNA was extracted from cells and real-time PCR was performed with *CKAP5*-specific primers. CT value obtained was used to calculate the mRNA copy number based on the equation obtained from the standard curve. **The data represents average Ct value ± SEM from 3 representative experiments (n=3).**

Supplementary Figure 2

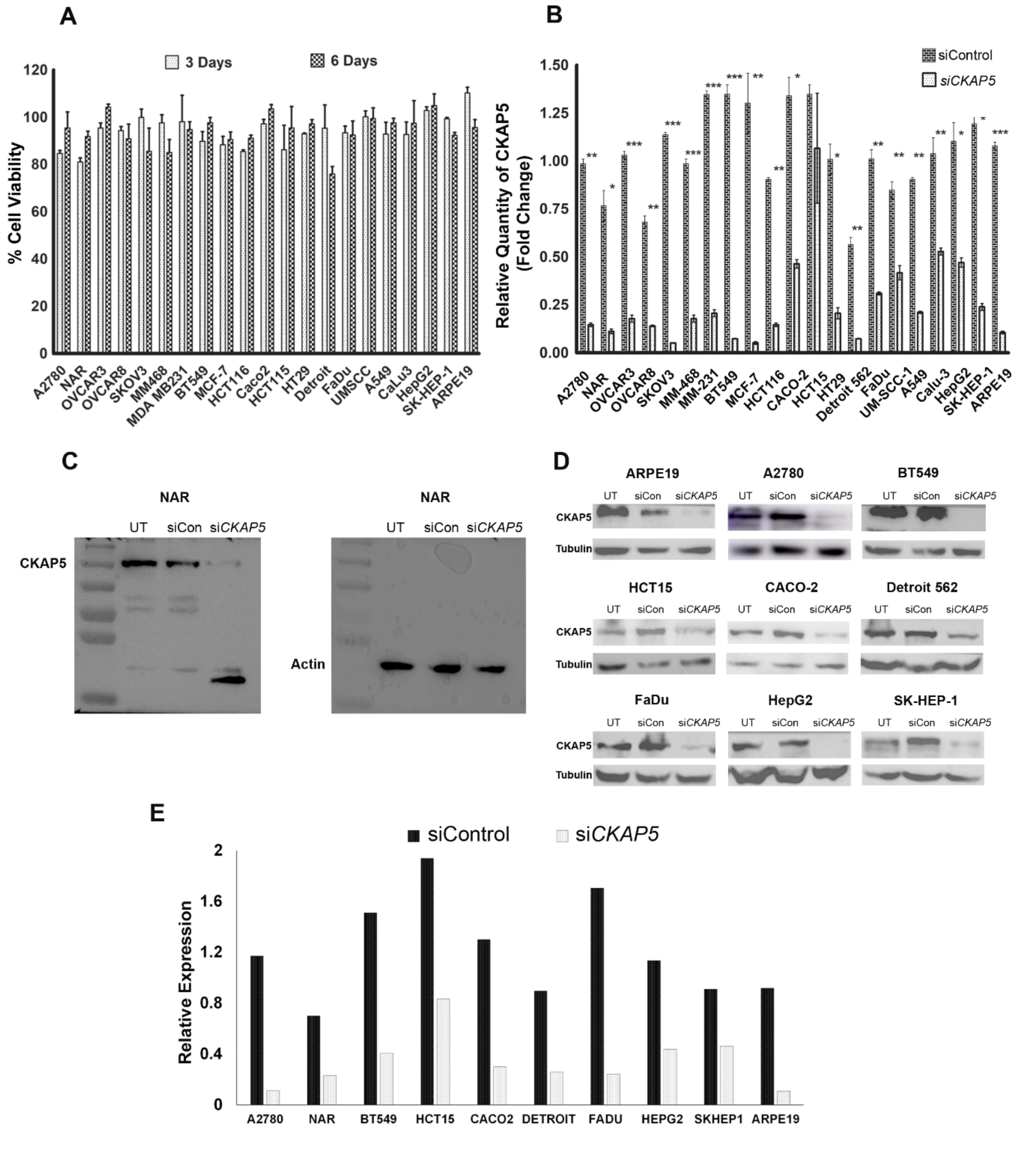

Supplementary Figure 2 LNP toxicity and *CKAP5* silencing efficiency across various solid cancer cell lines **A.** Toxicity of siCcontrol-LNPs across various cancer cell lines at a dose of 0.25µg/ml of particle incubation for 3 days and 6 days. **Data is represented as mean ± SEM from 3 representative experiments (n=3) and analysed by an unpaired t-test. B.** *CKAP5* silencing post 0.25µg/ml dose across various cancer cell lines. Real-time PCR was performed using *GAPDH* as an endogenous control. **Data is represented as mean fold change ± SEM from 2 representative experiments (n=3) and analysed by an unpaired t-test. * indicates p value less than 0.5, ** indicates p value less than 0.05 and *** indicates p value less than 0.005. C.** Western blot showing a complete gel result for CKAP5 specific binding of the anti-human

CKAP5 antibody in NAR cell's protein extract. The membrane was stripped and further probed for tubulin, which was used as a loading control. **D.** CKAP5 down regulation at the translational level post *CKAP5* silencing in a few representative sensitive as well as non-sensitive cell lines. Tubulin was used as a loading control. E. Densitometry analysis of *CKAP5* silencing as obtained from the western blot results.

Supplementary Figure 3

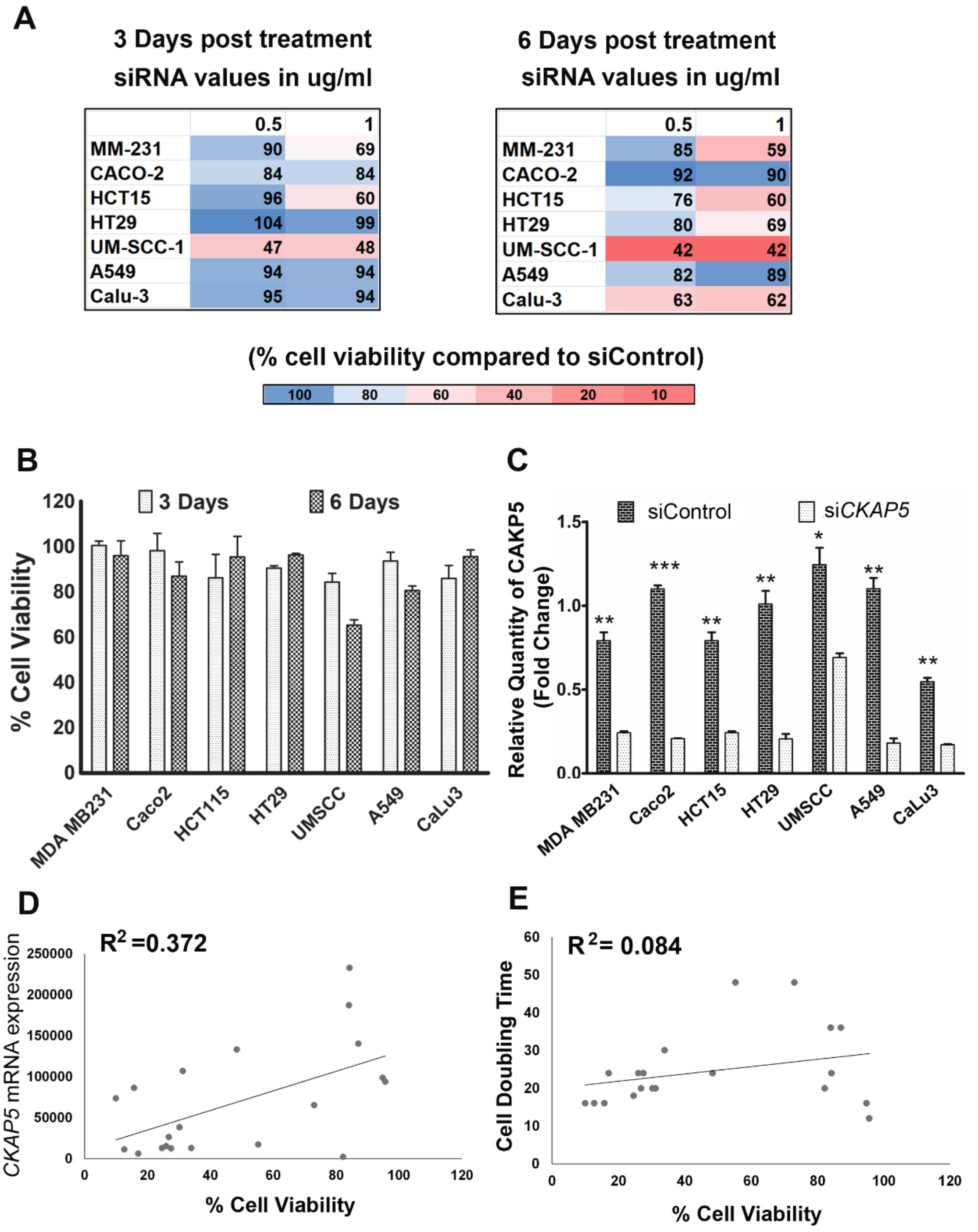

Supplementary Figure 3: Silencing, toxicity, and efficacy of LNPs at 1µg/ml dose in non-responsive cells. A. Effect of *CKAP5* knock-down on non-responsive cells at a dose of 1µg/ml for 3 days and 6 days post-LNP incubation. Data is represented as an average of 3 experiments with 3 technical repeats each time. B. Toxicity of control LNPs across various cancer cell lines at a 1µg/ml dose after 3 and 6 days. **Data is represented as mean ± SEM from 3 representative experiments (n=3) and analysed by an unpaired t-test.** C. Silencing efficiency of si*CKAP5*-LNPs in non-responsive cells at a dose of 1µg/ml as measured by real-time PCR. *GAPDH* was used as an endogenous control. **Data is**

represented as mean ± SEM from 3 representative experiments (n=3) and analysed by an unpaired t-test. * indicates p value less than 0.5, ** indicates p value less than 0.05 and * indicates p value less than 0.005.** D. Correlation of *CKAP5* expression with cell viability in various cell lines. mRNA copy numbers were plotted against cell viability at 6 days of si*CKAP5*-LNP incubation E. Correlation of cell doubling time with cell viability in various cell lines. Cell doubling time was plotted against cell viability at 6 days of si*CKAP5*-LNP incubation.

Supplementary Figure 4

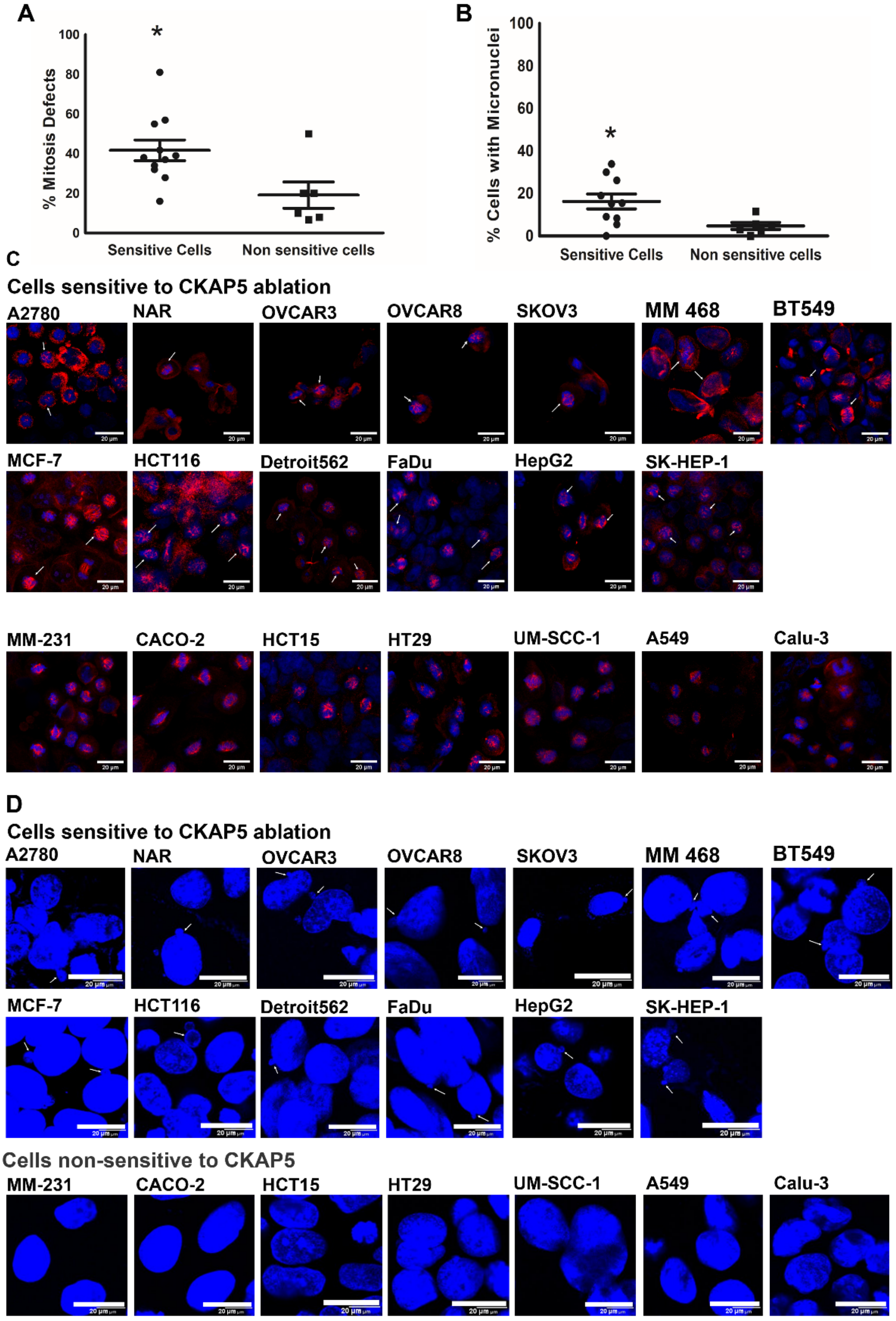

Supplementary Figure 4 Comparison of genetic instability in *CKAP5* siRNA sensitive versus non-sensitive cells **A**. Comparison of mitotic defects as measured by tubulin staining during mitosis in si*CKAP5* sensitive and non-sensitive cells. **Data is represented as average of % mitotic defects ± SEM from 50 mitotic events and analysed by unpaired t-test. ***

indicates p value of 0.02. **B**. Comparison of micronuclei formation as observed by DAPI staining in si*CKAP5* sensitive versus non-sensitive cells. **Data is represented as average of % cells with micronuclei formation ± SEM from 50 cells and analysed by unpaired t-test**. *** indicates p value of 0.03. C.** Representative confocal microscopic images of cells showing mitotic spindle status in 20 solid cancer cell types. Cells were stained for tubulin to detect microtubules and the nucleus was counterstained with DAPI. The scale bar represents 20 microns. Multicentric spindle are represented by white arrows. **D.** Representative confocal microscopic images of cells showing the presence of micronuclei in 20 solid cancer cell types. The cell nucleus was stained with DAPI and images were captured under a confocal microscope at 60 x magnification with 4x zoom. The scale bar represents 20 microns. Micronuclei are represented by white arrows

Supplementary Figure 5

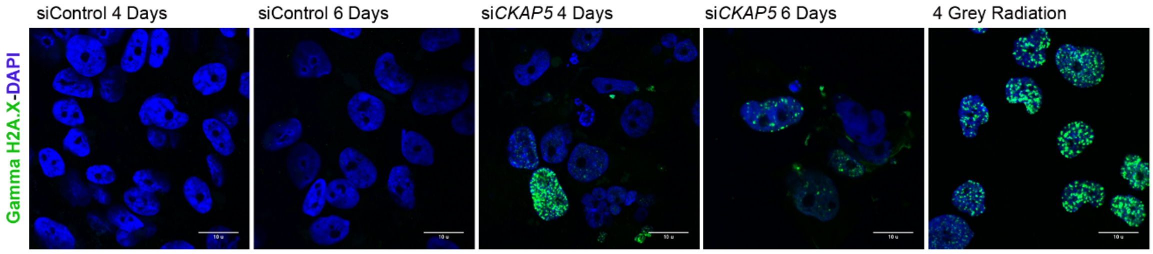

Supplementary Figure 5 Representative confocal microscope images for gamma H2A.X foci formation in control/si*CKAP5* LNP treated cells. Cells were stained for γH2A.X and the nucleus was counterstained with DAPI. The scale bar represents 10 microns.

Supplementary Figure 6

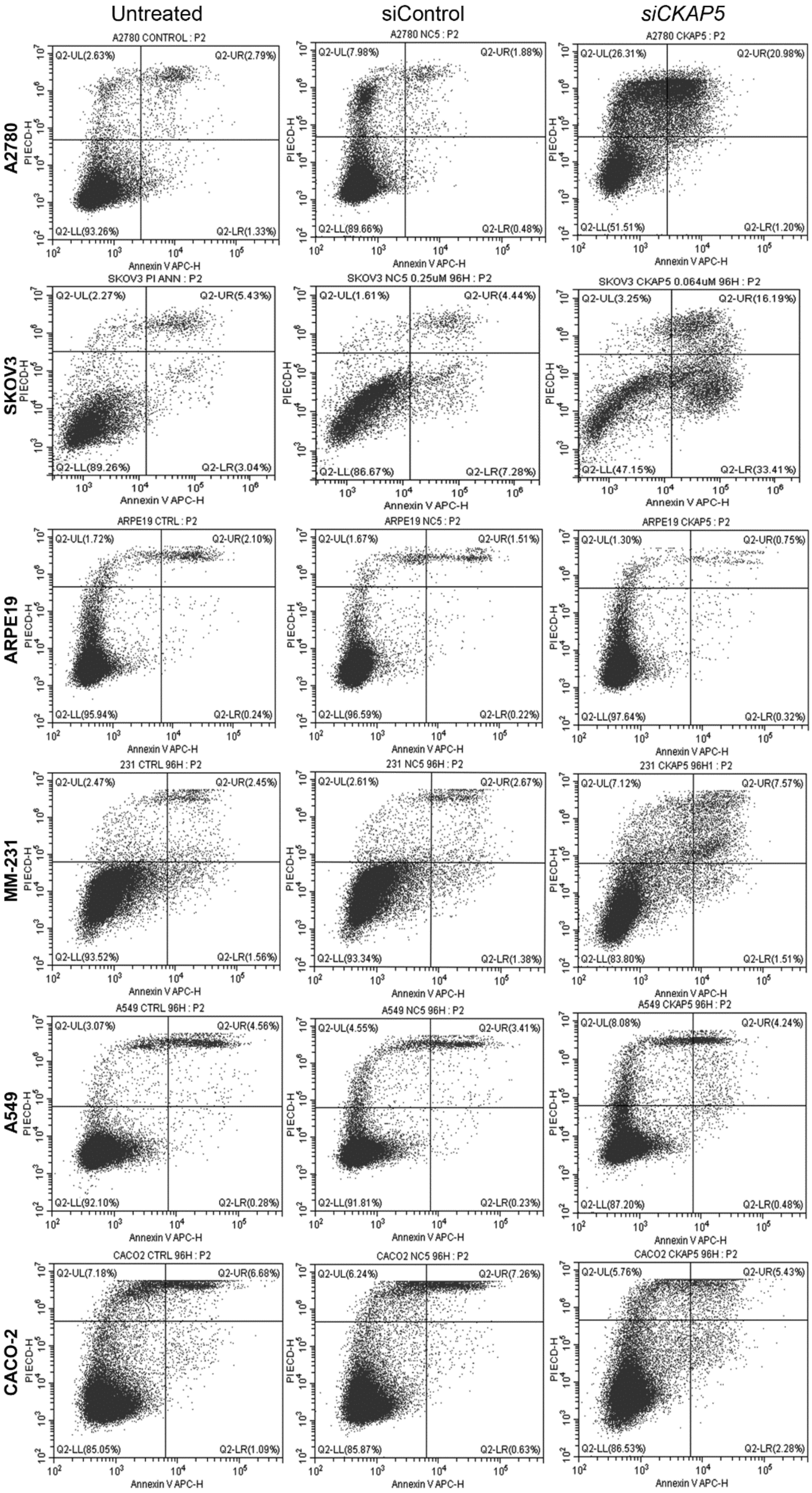

Supplementary Figure 6 Dot plot data of flow cytometric analysis for PI Annexin assay in CKAP5 sensitive and non-sensitive cell lines. The X-axis represents AnnexinV APC and Y-axis represents Propidium Iodide. Sensitive cell lines were incubated with 0.25µg/ml of siControl/si*CKAP5*-LNPs and non-sensitive cell lines were treated with 1µg/ml of siControl/siCKAP5-LNPs. The assay was performed 96 hours post particle incubation.

Supplementary Figure 7

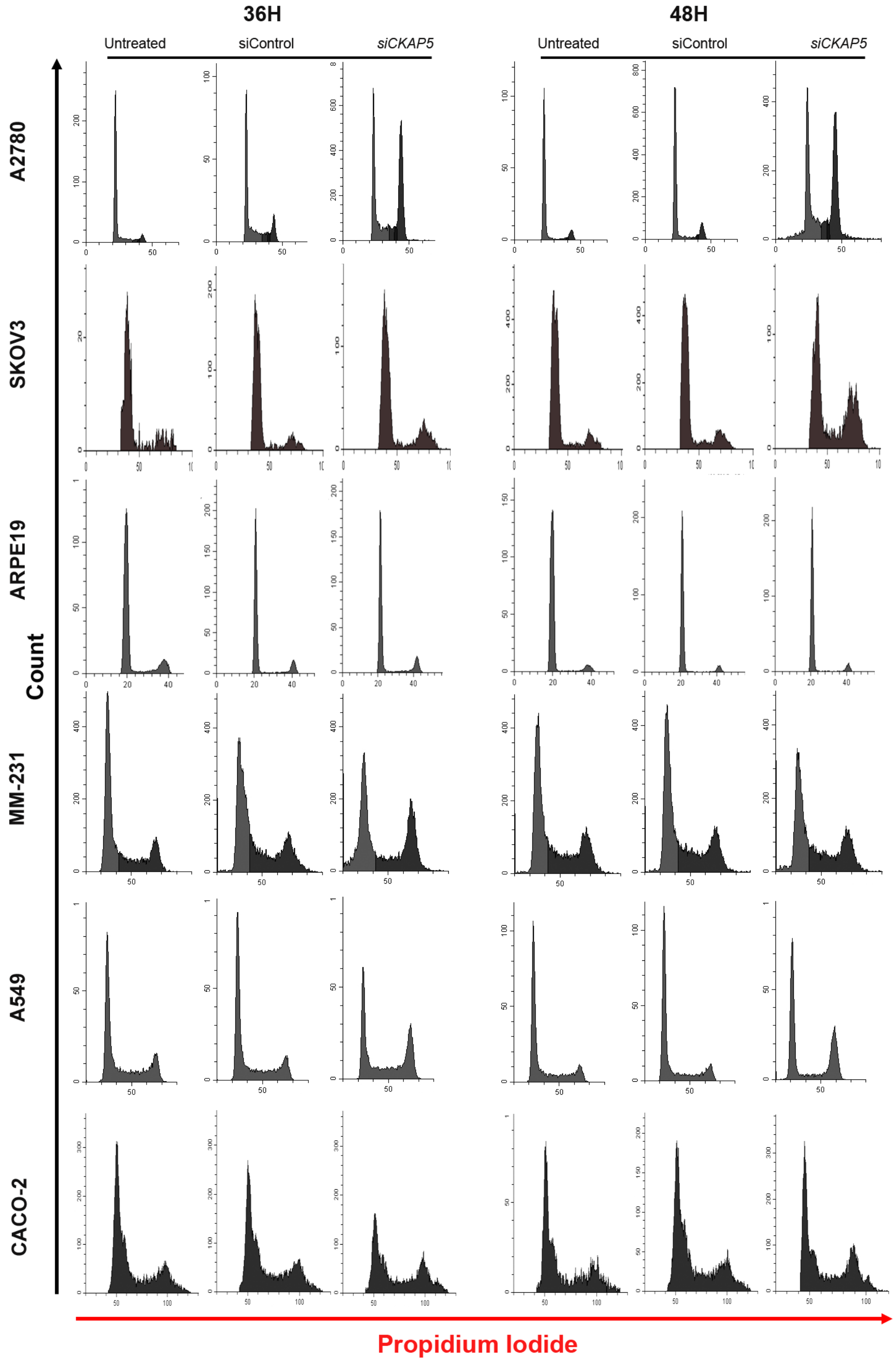

Supplementary Figure 7 Histogram data of flow cytometric analysis for cell cycle arrest in a few representative CKAP5 sensitive and non-sensitive cell lines. Sensitive cell lines were incubated with 0.25µg/ml of siControl/si*CKAP5*-LNPs and non-sensitive cell lines were treated with 1µg/ml of control/si*CKAP5*-LNPs. Cells were stained for propidium iodide after ethanol fixation at 36 and 48 hours of particle incubation**.**

Supplementary Figure 8

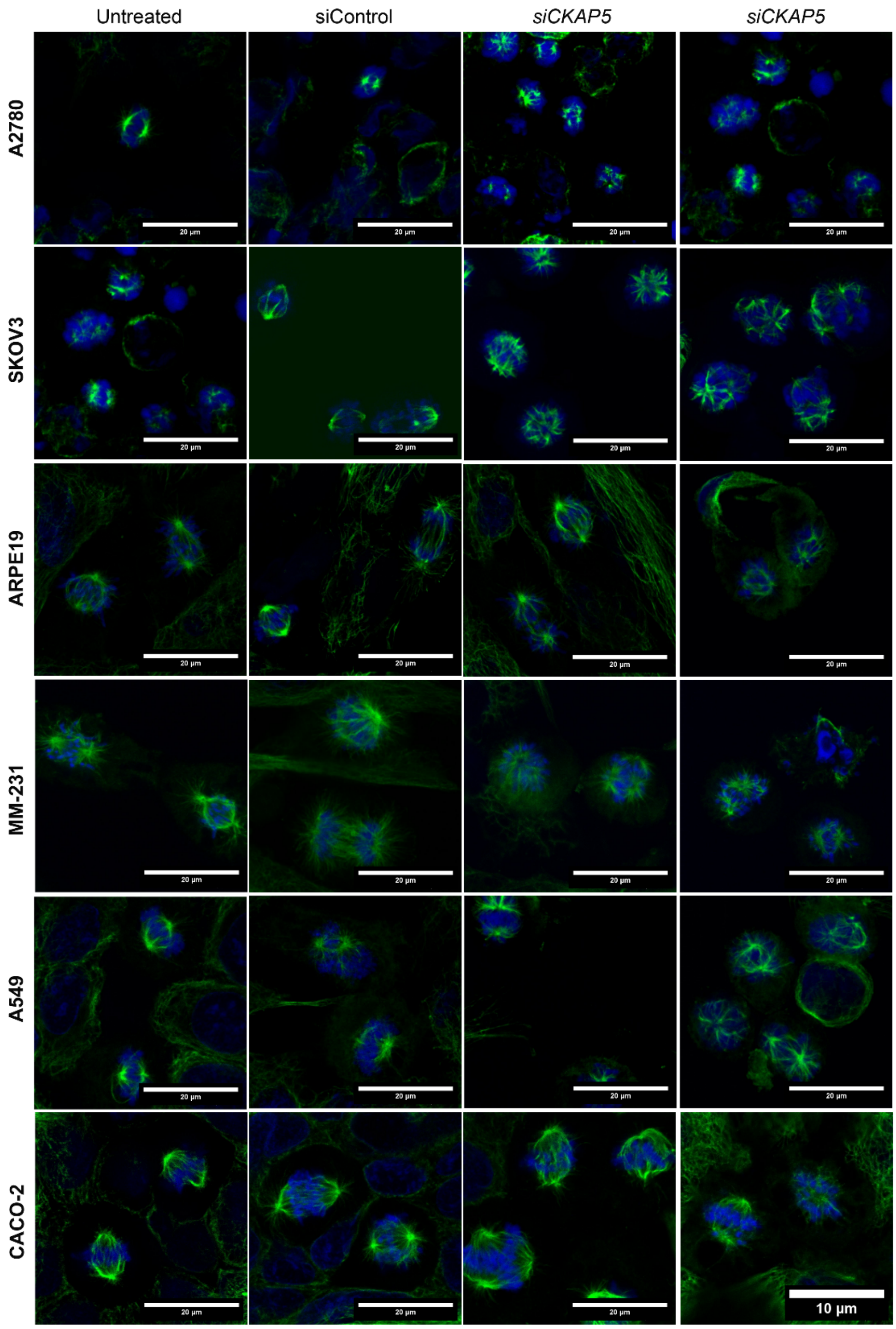

Supplementary Figure 8 Confocal microscopic images of representative cell lines showing mitotic spindle status in control and si*CKAP5* LNP treated cells. Cells were stained with tubulin antibody and the nucleus was counterstained with DAPI. Images were captured at 60x magnification with 2x zoom. The scale bar represents 10micron.

Supplementary Figure 9

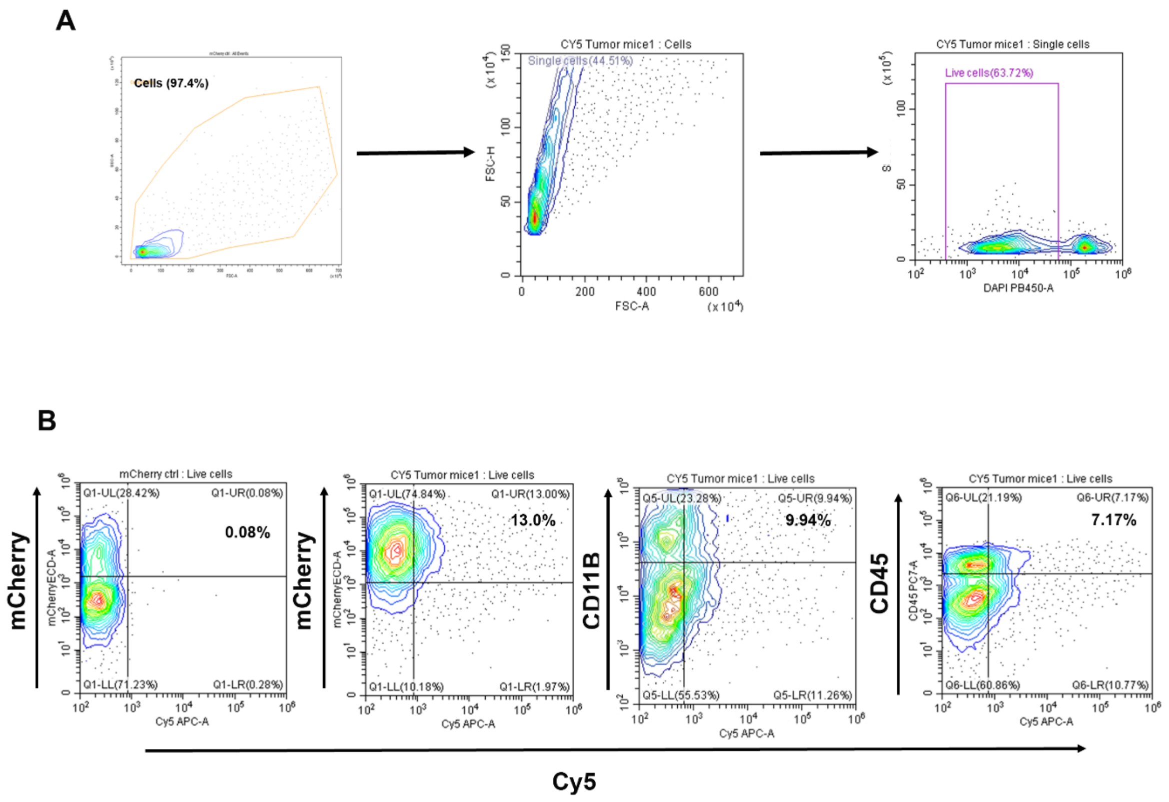

Supplementary Figure 9 Flow cytometry data representing biodistribution of Cy5 labelled LNPs in tumor tissue. A. Gating strategy used to identify single live cell population. B. Dot plot data representing CY5-LNP uptake by CD45 positive, CD11B positive, and *mCherry* positive tumors cells. Mice were injected with 1mg kg^{-1} of Cy5-LNPs and tumor tissue was extracted 2 hrs post-injection. Tumor tissue from untreated mice was used as a control. Tissue was processed to obtain single cells and stained for anti-CD45, anti-CD11B antibodies, and DAPI.

Supplementary Figure 10

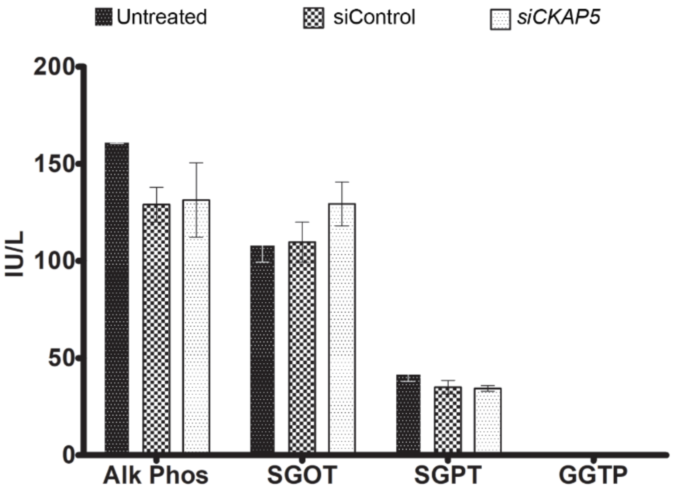

Supplementary Figure 10 Data representing liver enzyme levels in control/si*CKAP5*-LNPs treated mice. Mice serum was collected after 2 hours of control/si*CKAP5*-LNP treatment and subjected to liver enzyme tests. **Data represents mean ± SEM from 2 representative experiments (n=3).**

Table S1

LNP Conc (ug/ml)	0.0017	S.D.	0.0035	S.D.	0.007	S.D.	0.015	S.D.	0.03	S.D.	0.06	S.D.	0.12	S.D.	0.25	S.D.
A2780	100	+/-3.8	90	+/-3.01	70	+/-5.0	50	+/-5.9	37	+/-4.5	30	+/-4.5	28	+/-4.1	26	+/- 4.25
NAR	117	+/- 2.5	105	+/-5.5	92	+/-5.4	75	+/-6.3	66	+/-5.9	61	+/-7.8	53	+/-6.9	43	+/-7.3
OVCAR3	100	+/- 2.6	97	+/-1.7	103	+/-2.6	99	+/-8.4	84	+/-3.9	71	+/-6.3	66	+/-5.1	73	+/-4.3
OVCAR8	90	+/-8.1	86	+/-7.8	85	+/-7.5	72	+/-7.7	66	+/-3.4	64	+/-1.2	64	+/-1.0	56	+/-5.0
SKOV3	104	+/-5.6	106	+/-3.4	95	+/-7.1	79	+/-1.4	74	+/-2.5	70	+/-1.3	61	+/-1.7	56	+/-1.6
MM-468	87	+/-4.5	53	+/-6.7	27	+/-3.9	41	+/-5.5	39	+/-2.5	31	+/-6.7	22	+/-4.3	22	+/-3.8
MM-231	101	+/-2.5	106	+/-1.1	102	+/-3.9	103	+/-0.4	101	+/-0.8	102	+/-1.9	94	+/-1.2	97	+/-3.4
BT549	89	+/-4.9	91	+/-6.9	78	+/-6.5	58	+/-4.5	45	+/-7.7	38	+/-5.5	41	+/-3.8	48	+/-1.2
MCF-7	98	+/-3.4	93	+/-7.5	86	+/-6.3	81	+/-4.9	77	+/-4.5	71	+/-5.2	66	+/-4.5	57	+/-6.0
HCT116	28	+/-5.4	25	+/-6.5	26	+/-3.2	21	+/-5.4	21	+/-2.9	20	+/-4.3	20	+/-6.7	24	+/-5.9
CACO-2	104	+/-2.4	100	+/-2.2	93	+/-4.8	95	+/-5.0	90	+/-6.1	84	+/-4.9	76	+/-3.9	72	+/-2.8
HCT15	121	+/-2.3	111	+/-7.5	120	+/-7.2	115	+/-6.5	119	+/-5.1	110	+/-7.2	108	+/-5.9	111	+/-5.4
HT29	102	+/-4.9	104	+/-0.9	95	+/-2.3	106	+/-7.8	103	+/-6.7	104	+/-4.5	105	+/-8.7	101	+/-3.4
Detroit 562	85	+/-5.5	69	+/-7.8	68	+/-4.9	60	+/-3.2	50	+/-5.8	55	+/-3.7	51	+/-6.6		+/-2.5
FaDu	99	+/-2.3	92	+/-2.2	93	+/-4.6	87	+/-3.3	89	+/-3.7	80	+/-3.7	83	+/-3.8	86	+/-5.2
UM-SCC-1	109	+/-4.5	105	+/-6.6	110	+/-3.3	103	+/-5.8	100	+/-2.3	95	+/-5.5	96	+/-6.4	93	+/-4.6
A549	112	+/-5.5	104	+/-0.5	110	+/-3.8	97	+/-0.1	98	+/-5.0	100	+/-5.7	86	+/-1.1	84	+/-3.9
Calu-3	114	+/-0.8	109	+/-3.5	106	+/-1.06	102	+/-4.5	95	+/-3.7	91	+/-2.4	93	+/-0.6	97	+/-2.9
HepG2	71	+/-2.0	67	+/-4.1	55	+/-4.4	44	+/-4.3	45	+/-1.3	47	+/-3.4	46	+/-1.5	44	+/-2.9
SK-HEP-1	77	+/-7.4	65	+/-5.3	60	+/-3.4	57	+/-4.7	54	+/-3.3	50	+/-4.9	48	+/-7.8	48	+/-5.6
ARPE19	104	+/-6.7	102	+/-2.3	100	+/-5.3	99	+/-4.8	95	+/-4.9	95	+/-4.4	93	+/-6.4	92	+/-3.4

Cell viability in response to *CKAP5* knock-down 3 days post treatment. **Values are represented as mean of % viable cells as compared to siCcontrol-LNP treated cells ± SEM.**

Table S2

LNP Conc (ug/ml)	0.0017	S.D.	0.0035	S.D.	0.007	S.D.	0.015	S.D.	0.03	S.D.	0.06	S.D.	0.12	S.D.	0.25	S.D.
A2780	102	+/-1.2	101	+/-0.8	87	+/-2.2	68	+/-2.7	34	+/-2.3	22	+/-1.3	18	+/-0.8	16	+/-0.19
NAR	110	+/-0.8	110	+/-4.8	107	+/-6.1	98	+/-3.9	84	+/-7.3	63	+/-1.7	37	+/-7.8	27	+/-6.1
OVCAR3	94	+/-1.9	98	+/-1.4	99	+/-1.6	99	+/-1.4	97	+/-1.3	94	+/-2.1	74	+/-3.6	48	+/-3.0
OVCAR8	95	+/-0.3	98	+/-6.2	92	+/-5.2	77	+/-4.8	57	+/-1.1	46	+/-3.8	33	+/-4.7	25	+/-6.7
SKOV3	117	+/-5.9	113	+/-4.0	96	+/-6.8	78	+/-5.7	74	+/-2.6	61	+/-3.6	39	+/-4.7	34	+/-3.9
MM468	86	+/-1.2	51	+/-2.9	16	+/-0.9	18	+/-1.0	16	+/-0.98	14	+/-0.9	11	+/-0.6	10	+/-0.2
MDA MB231	98	+/-0.4	97	+/-2.4	97	+/-2.6	106	+/-0.5	105	+/-0.8	96	+/-1.9	95	+/-0.8	84	+/-1.9
BT549	99	+/-7.2	93	+/-5.1	87	+/-3.5	61	+/-5.5	30	+/-6.9	26	+/-9.4	26	+/-1.9	26	+/-4.7
MCF-7	100	+/-4.5	94	+/-5.0	79	+/-3.3	67	+/-3.2	65	+/-8.6	56	+/-7.5	44	+/-6.7	30	+/-4.8
HCT116	30	+/-5.5	19	+/-5.4	16	+/-3.4	13	+/-5.6	12	+/-4.5	11	+/-3.4	13	+/-4.8	13	+/-4.7
CACO-2	97	+/-5.1	101	+/-3.5	101	+/-4.6	95	+/-4.8	95	+/-4.8	89	+/-4.6	87	+/-4.0	84	+/-4.6
HCT15	102	+/-1.3	99	+/-4.1	101	+/-4.3	107	+/-3.2	102	+/-2.9	96	+/-2.2	90	+/-2.6	82	+/-3.6
HT29	104	+/-5.2	105	+/-2.5	101	+/-3.7	107	+/-0.4	101	+/-5.0	108	+/-9.0	111	+/-4.3	95	+/-5.1
Detroit 562	78	+/-3.7	63	+/-4.4	48	+/-2.8	36	+/-5.1	25	+/-3.4	22	+/-2.8	20	+/-5.7		+/-7.1
FaDu	97	+/-4.3	86	+/-4.5	69	+/-4.9	56	+/-4.3	46	+/-5.5	42	+/-4.2	35	+/-3.7	31	+/-4.3
UM-SCC-1	103	+/-1.9	104	+/-1.7	110	+/-3.2	92	+/-1.2	94	+/-1.9	76	+/-3.8	67	+/-1.1	55	+/-1.2
A549	105	+/-3.5	107	+/-3.4	111	+/-5.3	103	+/-2.8	102	+/-3.7	93	+/-3.8	98	+/-3.3	96	+/-2.2
Calu-3	105	+/-1.5	102	+/-6.8	100	+/-4.3	90	+/-4.1	85	+/-5.3	78	+/-4.6	76	+/-3.3	73	+/-3.0
HepG2	55	+/-2.9	45	+/-1.6	33	+/-0.6	28	+/-1.2	24	+/-1.5	22	+/-0.85	19	+/-1.8	17	+/-1.4
SK-HEP-1	60	+/-2.4	58	+/-5.5	55	+/-3.5	48	+/-5.5	40	+/-6.3	35	+/-2.2	30	+/-3.4	28	+/-4.8
ARPE19	102	+/-2.7	100	+/-1.8	98	+/-4.0	98	+/-3.6	99	+/-3.2	94	+/-3.2	95	+/-3.5	87	+/-1.6

Cell viability in response to *CKAP5* knock-down 6 days post treatment. **Values are represented as mean of % viable cells as compared to siCcontrol-LNP treated cells ± SEM.**

Table S3

Cell Line	Medium	FBS	Pen-Strept	L-Glutamine	Supplements
**A2780**	RPMI	10%	1%	1%	
**NAR**	RPMI	10%	1%	1%	
**OVCAR3**	RPMI	10%	1%	1%	
**OVCAR8**	RPMI	10%	1%	1%	
**SKOV3**	McCoy's5A	10%	1%	1%	
**MM468**	RPMI	10%	1%	1%	
**MDA MB231**	DMEM	10%	1%	1%	
**BT549**	RPMI	10%	1%	1%	
**MCF-7**	EMEM	10%	1%	1%	Insulin (0.01mg/ml)
**HCT116**	RPMI	10%	1%	1%	
**Caco2**	EMEM	10%	1%	1%	
**HCT15**	RPMI	10%	1%	1%	
**HT29**	McCoy's5A	10%	1%	1%	
**Detroit**	DMEM	10%	1%	1%	NEAA 1%, Na-Pyruvate 1%
**FaDu**	DMEM	10%	1%	1%	NEAA 1%
**UMSCC**	DMEM	10%	1%	1%	NEAA 1%
**A549**	RPMI	10%	1%	1%	
**CaLu3**	DMEM	10%	1%	1%	
**HepG2**	EMEM	10%	1%	1%	
**SK-Hep1**	EMEM	10%	1%	1%	

List of cell lines and respective cell culture media used in the study

Table S4

Gene	Fwd Primer	Rev Primer
AURKB	CAGAGAGATCGAAATCCAGGC	CCTTGAGCCCTAAGAGCAGAT
BUB1	GAGTGATATCTTCAGCTTGTG	AACAACCTGCTCAACATCAAC
BUB1B	ACGTTATTAGAAAGAGCTGTAG	CATATCCAAAGGCTCATTGC
CCNA2	GCATGTCACCGTTCCTCCTT	GGGCATCTTCACGCTCTATTT
CDC20	CCTCTGGTCTCCCCATTAC	ATGTGTGACCTTTGAGTTCAG
MAD2L1	GCGCGTGCTTTTGTTTGTG	AGTAAAGGTTTCAGATGGATAT
MAD2L2	TGCATCTCATCCTCTACGTG	TCCTGGATATACTGATTCAGC
NDC80	CCTCTCCATGCAGGAGTTAAGA	GGTCTCGGGTCCTTGATTTTCT
NEK2	CATTGGCACAGGCTCCTAC	GAGCCATAGTCAAGTTCTTTCCA
TACC3	CCTCTTCAAGCGTTTTGAGAAAC	GCCCTCCTGGGTGATCCTT
TTK	AATGCTGGAAATTGCCCTGC	GAACCGGAAAATGATTCTTGG

List of genes studied for real time PCR and the respective primer sequence

Movie S1

Live cell kinetics to follow cell cycle in Tubulin.GFP-H2B.mCherry labelled ***untreated*** NAR cells

Movie S2

Live cell kinetics to follow cell cycle in Tubulin.GFP-H2B.mCherry labelled ***siControl treated*** NAR cells

Movie S3

Live cell kinetics to follow cell cycle in Tubulin.GFP-H2B.mCherry labelled ***siCKAP5 treated*** NAR cells

Movie S4

Live cell imaging to follow tubulin + ends in ***siControl*** treated EB3.eGFP labelled NAR cells

Movie S5

Live cell imaging to follow tubulin + ends in ***siCKAP5*** treated EB3.eGFP labelled NAR cells
